# Cancer type-specific adverse events of immune checkpoint inhibitors: A systematic review and meta-analysis

**DOI:** 10.1016/j.heliyon.2024.e41597

**Published:** 2025-01-02

**Authors:** Xuhui Tong, Rong Tang, Jin Xu, Wei Wang, Qiong Du, Si Shi, Xianjun Yu

**Affiliations:** a Department of Pancreatic Surgery, Fudan University Shanghai Cancer Center, Shanghai, China; b Department of Oncology, Shanghai Medical College, Fudan University, Shanghai, China; c Shanghai Pancreatic Cancer Institute, Shanghai, China; d Pancreatic Cancer Institute, Fudan University, Shanghai, China; e Department of Pharmacy, Fudan University Shanghai Cancer Center, Shanghai, China

## Abstract

**Background:**

The distribution of adverse events (AEs) triggered by immune checkpoint inhibitors (ICIs) across different cancer types has never been demonstrated.

**Methods:**

Randomised controlled trials exclusively assessing ICI monotherapy in cohorts of over 100 patients were considered. Our primary outcome was a comprehensive summary of the distribution of all-grade treatment-related adverse events (TRAEs) as well as serious TRAEs (CTCAE grade 3 or higher) across different malignancies. The study is registered with PROSPERO CRD42023387934.

**Findings:**

75 trials that enrolled over 100 patients were included. While investigating the incidence of each TRAE across various cancers, we found special linkages existed between certain TRAEs and particular cancer types. In anti-PD-1 monotherapy group, melanoma patients experienced the most frequent fatigue (31.1 %, 95 % CI 29.7%–32.5 %); the incidences of severe pneumonitis and other respiratory disorders were highest in Hodgkin lymphoma (4.1 %, 95 % CI 1.5%–8.6 %; 4.1 %, 95 % CI 1.5%–8.6 %, respectively). Among individuals undergoing single-agent anti-PD-L1, higher frequency of all-grade pruritus occurred in 19.0 % of renal cell carcinoma (RCC) patients (95 % CI 15.2%–23.2 %), and the highest probability of developing other severe musculoskeletal disorders was observed in patients with RCC (6.2 %, 95 % CI 4.0%–9.0 %). In anti-CTLA-4 monotherapy, the incidences of both all-grade and severe diarrhea occurred most frequently in prostate cancer patients (41.9 %, 95 % CI 37.9%–47.9; 14.8 %, 95 % CI 11.5%–18.7 %, respectively).

**Interpretation:**

This is the first comprehensive study addressing the distribution of various TRAEs across cancer types. Our research emphasizes the significance of considering cancer-specific TRAEs when using ICIs for treatment.

## 1 Introduction

Over the past decade, the introduction of immune checkpoint inhibitors (ICIs) has profoundly reshaped the therapeutic landscape for a wide spectrum of cancer types, especially those in advanced stages [1]. ICIs function by alleviating inhibitory barriers on T cells, such as programmed cell death 1 (PD-1) and cytotoxic T lymphocyte-associated protein 4 (CTLA-4), or by mitigating coinhibitory signals on tumor cells and antigen-presenting cells, like programmed cell death ligand-1 (PD-L1). This mechanism triggers robust activation of the body's own immune system, leading to effective elimination of malignant cells [2]. ICIs have gained approval for the treatment of a wide range of solid tumors and hematologic malignancies, encompassing both metastatic and adjuvant treatment contexts [3,4]. Furthermore, strategies combining ICIs with other anticancer drugs have also been proposed [5]. However, we should not ignore the adverse events (AEs) that may be triggered by treatments [6,7]. Complicated AEs continue to be a significant concern, as they may lead to treatment termination or even death from AEs [8].

Treatment-related AEs (TRAEs), which can affect most organ systems, have been observed in many patients undergoing checkpoint blockade monotherapy [9]. However, heterogeneity exists in the profile and severity of TRAEs among patients, even when the same drug is administered [10]. No previous study has compared the profiles of TRAEs across various malignancies. There are still no clear recommendations for specific screening programs or prevention efforts for different cancer populations [11].

Therefore, we designed a meta-analysis to examine all-grade TRAEs and serious TRAEs across various cancers treated with ICI monotherapy. We only extracted data from studies utilizing single-agent ICI therapy to clarify whether the incidence of certain TRAEs is specifically related to cancer type. We also compared the occurrence of TRAEs in cancer patients with adjuvant (including neoadjuvant) ICI therapy versus nonadjuvant ICI therapy. Our research can help with the development of TRAE screening and monitoring profiles for various cancer patients and provide TRAE-related evidence for the selection of ICI regimens.

## 2 Methods

The results are reported following the PRISMA guidelines and are prospectively registered in PROSPERO (2023 CRD42023387934) [12].

### 2.1 Data sources and searches

We conducted searches in the MEDLINE, Embase, Web of Science, and Cochrane Central databases for English-language studies published between January 1, 2012, and December 31, 2022. The main search terms included cancer (carcinoma, neoplasm), ICIs (anti-PD-1, anti-PD-L1, and anti-CTLA-4), and specific ICI drug names (nivolumab, pembrolizumab, and avelumab, atezolizumab, ipilimumab, tremelimumab). Detailed search strategies can be found in Table S1. Additionally, we searched the reference lists of the included studies to identify additional eligible records.

### 2.2 Eligibility criteria

The eligibility criteria were as follows: [1] randomised controlled trials (RCTs) that enrolled more than 100 patients with active cancer of solid organs or haematological malignancies; [2] articles in which participants received ICI drug monotherapy (irrespective of treatment history, dosage and duration); and [3] articles reporting the categorized incidences or tabular data of all-grade (Common Terminology Criteria for adverse events [CTCAE] grade 1 to 5) TRAEs with or without serious (CTCAE grade 3 to 5) TRAEs. Studies were excluded if they were reviews, conference abstracts, comments, editorials, meta-analyses, or cost effectiveness analyses. Studies that did not clarify the exact cancer type and/or cancer location were also excluded. If several studies reported outcomes of overlapping patients, we only included the one with the most detailed data on all-grade TRAEs.

Two authors (TXH and TR) independently screened the titles and abstracts of the retrieved studies. Subsequently, they evaluated the full texts of the studies to determine inclusion independently. Any disagreements between the authors were resolved by consensus with a third senior author (SS).

### 2.3 Data extraction

A standardized form for extracting data was designed and agreed upon by the research team. The following data points were obtained: study features (authors, year, trial phase, trial name, CTCAE version), patient characteristics (cancer type, number of participants, age, gender ratio, ethnicity), treatment details (treatment arms, drug name, drug target, dose, mode of administration, treatment history, PD-L1 expression), and study outcomes (number of patients with each specific all-grade TRAE, serious TRAEs, and overall incidence of all-grade TRAEs).

Both authors (TXH and TR) independently reviewed the text, supplemental materials, and relevant information on the Clinical Trial website to ensure that the data were comprehensive.

### 2.4 Risk of bias assessment

Two authors (TXH and TR) independently assessed the risk of bias in RCTs based on the original study design. The assessment was conducted using the Cochrane risk of bias tool, wherein each tool item, including random sequence generation, allocation concealment, participant and staff blinding, outcome assessment blinding, insufficient outcome data, selective reporting, and other bias, received a low, unclear, or high-risk bias evaluation [13]. The risk categorization for all included studies was determined based on the item with the highest risk among the seven evaluated items. Disagreements were resolved through discussion with a third senior author (SS). Detailed information on the risk of bias assessments is provided in Supplementary Material 1.

### 2.5 Data analysis

We employed random effects models for meta-analysis, weighting the models based on the number of patients with each type of cancer. All models were fitted using restricted maximum likelihood estimation with a classic continuity correction of 0.5 for zero cells and their corresponding sample sizes. The collected data were used to evaluate and compare the incidence rates of each TRAE across cancer types. Incidence rates for each TRAE are presented, stratified by cancer type and ICI target class, along with 95 % confidence intervals (95 % CI). We employed heatmap plots to present the data for each analysis group, offering a visual analysis of studies assessing all-grade TRAEs and serious TRAEs. Subgroup analyses were performed based on whether cancer patients were receiving PD-1 inhibitor or PD-L1 inhibitor therapy, the different treatment approaches (adjuvant/neoadjuvant and nonadjuvant therapy), and patients with varying pathological types of the same cancer. Additionally, we conducted a comparative analysis of TRAEs arising from different types of ICIs, specifically anti-PD-1 vs. anti-PD-L1, anti-CTLA-4 vs. anti-PD-1, and anti-CTLA-4 vs. anti-PD-L1, within the same cancer type.

We employed the I^2^ statistic to examine heterogeneity among the studies based on the proportion of patients with TRAEs among the total number at risk. To evaluate publication bias, we used a classic funnel plot, and we also conducted Egger's test to assess the log odds of all-grade events in studies involving the same ICI target (number of studies included ≥3). All analyses were conducted in R software (version 4.3.0).

### 2.6 Patient and public involvement

Patients, caregivers, or members from the public were not engaged in the study's production directly, as it is a meta-analysis based on previously published research. Nevertheless, the prime objective of this study was to foster a more comprehensive understanding of cancer-specific adverse events resulting from ICI treatment. We aspire to facilitate well-informed choices regarding ICI medications, improve patient management, and provide insights for making informed decisions about treatment regimens.

## 3 Results

A total of 6,098 studies published between January 1, 2012, and December 31, 2022, were initially retrieved from the databases. Four additional records added from reference lists of eligible full text articles were identified. After screening and assessing the eligibility of the studies, 75 RCTs involving 26,277 patients were ultimately included for the analysis of TRAEs (Table S2). Most of the included studies (66 out of 75) were phase III RCTs. After excluding gender-exclusive cancer types (e.g., prostate cancer in men and cervical cancer in women), the mean proportion of male participants among the included studies was 70.40 %. In terms of the racial composition of participants, there was a higher proportion of White or Caucasian patients compared to other racial groups, with percentages of 70.33 % for White or Caucasian, 24.59 % for Asian, 1.56 % for Black or African American, 0.36 % for American Indian or Alaskan Native, 0.14 % for Native Hawaiian or Other Pacific Islander, and 3.02 % for other racial backgrounds (P < 0.001) (Table S2). Table S2 presents the key characteristics of the included patients. The included studies examine practically all types of TRAEs across 16 different cancer types. We grouped the included studies based on targeted immune checkpoint type: anti-CTLA-4, anti-PD-1, and anti-PD-L1. Table S2 shows the number of people using different ICI drugs (PD-1 inhibitor: 18,854 patients, PD-L1 inhibitor: 4,858 patients, CTLA-4 inhibitor: 2,565 patients).

TRAEs from 12 organ systems (including metabolism disorders) were reported in the included studies, and details about the assessed AEs are presented in supplemental material 2. All-grade TRAEs were reported in 75 studies, which included a total of 58 arms of anti-PD-1 monotherapy, 17 arms of anti-PD-L1 monotherapy, and 7 arms of CTLA-4 inhibition monotherapy. The overall incidence of all-grade TRAEs for anti-PD-1 monotherapy, anti-PD-L1 monotherapy, and CTLA-4 inhibition therapy was 69.1 % (95 % CI 66.4%–71.8 %; I2 = 95 %), 66.1 % (95 % CI 61.2%–71.0 %; I2 = 93 %), and 82.4 % (95 % CI 73.9%–90.9 %; I2 = 98 %), respectively.

**Fig. 1:**
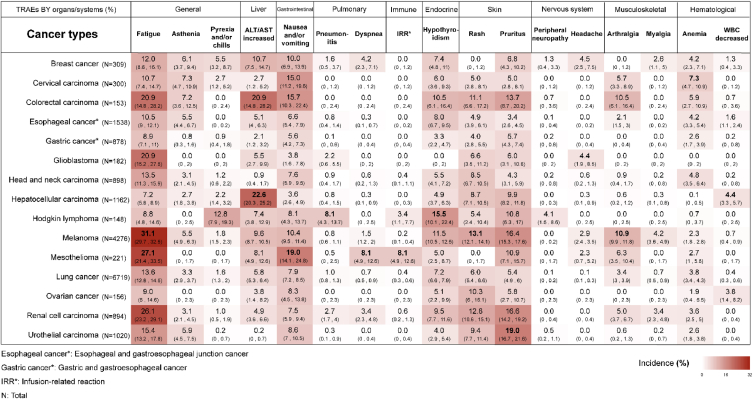
Incidence rate (95 % CI) of all-grade TRAEs in cancer patients treated with single-agent anti-PD-1.

Among cancer patients receiving PD-1 inhibitor monotherapy (Fig. 1), treatment-related fatigue was more likely to occur in patients with melanoma (31.1 %, 95 % CI 29.7%–32.5 %). Treatment-related pyrexia (and/or chills), pneumonitis, and hypothyroidism were more likely to be observed in Hodgkin lymphoma patients (12.8 %, 95 % CI 7.9%–19.3 %; 8.1 %, 95 % CI 4.3%–13.7 %; and 15.5 %, 95 % CI 10.1%–22.4 %, respectively). Furthermore, treatment-related musculoskeletal AEs, such as arthralgia and myalgia, exhibited a higher likelihood of occurrence in melanoma patients (10.9 %, 95 % CI 9.9%–11.8 %; 4.2 %, 95 % CI 3.6%–4.9 %, respectively). Melanoma patients reported the greatest percentage of rash incidence during anti-PD-1 therapy (13.1 %, 95 % CI 12.1%–14.1 %), whereas nearly one-fifth of patients with urothelial carcinoma reported experiencing pruritis (19.0 %, 95 % CI 16.7%–21.6 %). In addition, mesothelioma patients had the highest rates of treatment-related nausea and/or vomiting (19.0 %, 14.1%–24.8 %), dyspnea (8.1 %, 95 % CI 4.9%–12.6 %) and infusion-related reactions (IRRs) (8.1 %, 95 % CI 4.9%–12.6 %).

**Fig. 2:**
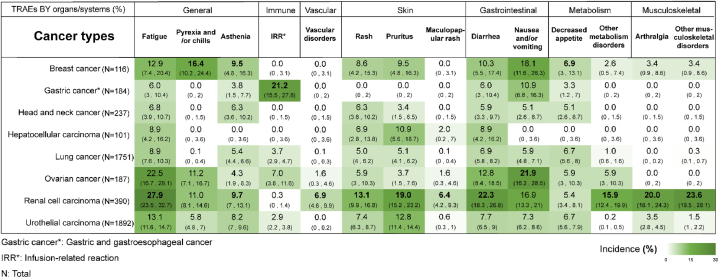
Incidence rate (95 % CI) of all-grade TRAEs in cancer patients treated with single-agent anti-PD-L1.

There were 8 kinds of malignancies analyzed in the PD-L1 inhibitor group (Fig. 2). Patients with renal cell carcinoma reported the greatest incidence rates of vascular problems associated with therapy (6.9 %, 95 % CI 4.6%–9.9 %), asthenia (9.7 %, 95 % CI 7%–13.1 %), and fatigue (27.9 %, 95 % CI 23.5%–32.7 %). Thirty-nine out of 184 gastric and gastroesophageal carcinoma patients reported IRR (21.2 %, 95 % CI 15.5%–27.8 %), and eighty-seven renal cell carcinoma patients experienced treatment-related diarrhea (22.3 %, 95 % CI 18,3%–26.8 %) after PD-L1 inhibitor treatment. Arthralgia and other musculoskeletal diseases attributed to anti-PD-L1 medication were more common among renal cell carcinoma patients than among individuals with other cancer types (20.0 %, 95 % CI 16.1%–24.3 %; 23.6 %, 95 % CI 19.5%–28.1 %, respectively). Additionally, patients with renal cell carcinoma had the highest frequency of treatment-related pruritus (19.0 %, 95 % CI 15.2%–23.2 %), and mesothelioma patients had the highest incidence of treatment-related nausea and/or vomiting (21.9 %, 95 % CI 16.2%–28.9 %).

**Fig. 3:**
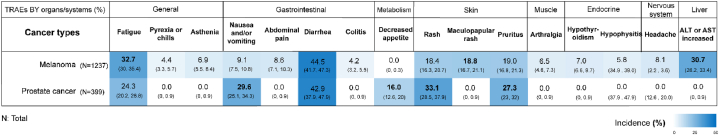
Incidence rate (95 % CI) of all-grade TRAEs in cancer patients treated with single-agent anti-CTLA-4.

In the CTLA-4 monotherapy group, RCTs about melanoma and prostate cancer using ipilimumab at 10 mg/kg of body weight were compared for their distribution within the same TRAE category. The heatmap in Fig. 3 shows that fatigue occurs in 26.2 % of melanoma patients who received anti-CTLA-4 therapy (95 % CI 24.4%–28.1 %). However, problems in the gastrointestinal, metabolic, and cutaneous systems were more commonly observed in patients with prostate cancer. For instance, 41.9 % of 399 prostate cancer patients reported diarrhea due to their therapy (95 % CI 37.9%–47.9), and 132 prostate cancer patients reported rash problems (33.1 %, 95 % CI 28.5%–37.9 %).

Differences have also been observed between TRAE profiles of PD-1 antibodies and PD-L1 antibodies [14]. And our analysis validated that the risk ratios (RR) for the safety profiles of anti-PD-1 therapy differed from those of anti-PD-L1 therapy in various cancer types (see Figs. S7, S8, and S9).

Among lung cancer patients, monotherapy with a PD-L1 inhibitor was associated with a significantly reduced risk of overall TRAEs compared with anti-PD-1 therapy (RR 1.18, 95 % CI 1.12–1.25, P < 0.0001). In particular, the anti-PD-1 subgroup exhibited a higher incidence of hyperthyroidism and renal disorders, whereas anti-PD-L1 therapy was associated with an increased risk of IRRs (Fig. S7). In renal cell carcinoma patients, the use of anti-PD-L1 is significantly associated with an increased risk of all-grade TRAEs compared to anti-PD-1 (RR 0.45, 95 % CI 0.42–0.48, P < 0.0001), as shown in Fig. S8. Interestingly, PD-1 inhibitor therapy was correlated with an increased incidence of IRR, while in contrast, almost all TRAE types were more common in the anti-PD-L1 treatment arm. There was no significant difference observed in urothelial carcinoma patients (RR 1.04, 95 % CI 0.97–1.12, 0.2815) (Fig. S9). Thyroid and renal disorder occurrences were correlated with anti-PD-1, and incidences of IRR, hepatobiliary disorders and pyrexia (or chills) were more correlated with anti-PD-L1 therapy.

## 4 Discussion

### 4.1 Principal findings

**Fig. 4:**
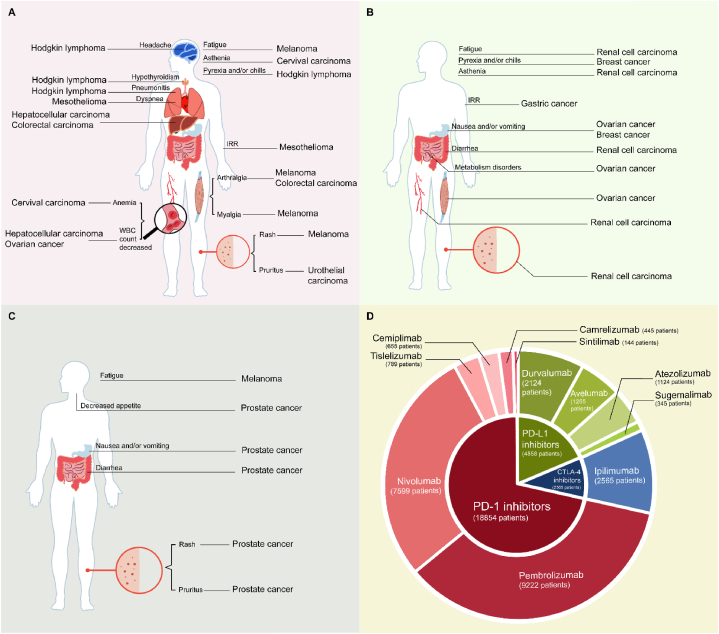
All-grade TRAEs had a much greater prevalence in specific cancer types. By Figdraw. A. Anti-PD-1 single agent group. B. Anti-PD-L1 single agent group. C. Anti-CTLA-4 single agent group. D. Pie chart of the proportion of patients with different ICI single-agent therapies included in this study.

This meta-analysis conducted a comprehensive comparison of AE incidences associated with ICI monotherapy across 16 different cancer types. As shown in Fig. 4, specific cancer types exhibited a significantly higher prevalence of certain TRAEs when stratified by the utilization of different checkpoint blockade therapies (anti-PD-1, anti-PD-L1, and anti-CTLA-4).

The predominant portion of study participants received PD-1 inhibitor therapy, and within this group, distinct patterns emerged (Fig. 4A). For instance, melanoma patients demonstrated a notably higher likelihood of experiencing all-grade treatment-related fatigue and arthralgia; Hodgkin lymphoma patients exhibited elevated incidence rates of pneumonitis and endocrine disorders; ovarian cancer patients exhibited increased incidence rates of severe maculopapular rash, pruritus, and hypothyroidism; and cervical cancer was linked to a higher incidence of serious-grade anaemia. In addition, in the anti-PD-L1 group, higher incidences of all-grade pruritus, diarrhea, and musculoskeletal disorders occurred in renal cell carcinoma; gastric and gastroesophageal carcinoma patients experienced a higher incidence of all-grade IRR; severe hypertension and metabolism disorders were more prevalent in renal cell carcinoma patients; and gastric cancer patients experienced a higher frequency of ALT/AST elevation in blood (Fig. 4B). Furthermore, significant disparities also exist in the types and frequencies of TRAEs among patients with the same cancer when treated with PD-1 antibodies versus PD-L1 antibodies (Figs. S7–S9). In the CTLA-4 inhibitor treatment group, both all-grade and serious-grade nausea, rash, and appetite loss were more prevalent in prostate cancer patients than in melanoma patients (Fig. 4C). The composition of ICIs by drug classes in the included studies is displayed in Fig. 4D.

### 4.2 Potential underlying mechanisms

The precise mechanisms underlying the higher prevalence of specific TRAEs in certain cancer types remain unclear. One plausible explanation is that autoreactive T cells, stimulated by ICI treatment, might migrate to physiological locations where tumour antigens are expressed, inadvertently targeting nontumour tissue [15]. For example, there was a higher frequency of vitiligo in patients with melanoma in anti-PD-1 therapy (Fig. 1) and anti-CTLA-4 therapy (Fig. 3). This situation is thought to be associated with T cells that have the capability to target both melanocytes and melanoma cells expressing tissue antigens, including melanocyte lineage-specific antigen (GP100), tyrosinase, and MART-1 [16,17]. Similarly, the incidence of pneumonitis was notably higher among lung cancer patients than among melanoma patients and has been associated with the infiltration of autoreactive napsin A-specific T lymphocytes. These T cells are responsible for both tumour cell destruction and the initiation of lung inflammation [18].

As depicted in supplemental material 2, individuals with Hodgkin lymphoma exhibit the highest prevalence of myocarditis associated with PD-1 inhibition, surpassing other malignancies. Previous research has established that survivors of Hodgkin lymphoma treated during adolescence or adulthood face an elevated risk of various cardiovascular diseases [19]. Moreover, multiple studies have reported an elevated risk of cardiovascular morbidity and mortality among Hodgkin lymphoma patients [[19], [20], [21]]. It is believed that the cardiac disorders related to ICI therapy are likely linked to T-cell-mediated responses targeting cardiac antigens. Additionally, several potential risk factors, such as treatment-induced skeletal myositis and certain treatment regimens involving dual ICIs, may contribute to adverse cardiac reactions [22]. Furthermore, traditional risk factors for heart disease, such as a history of smoking, hypertension, dyslipidaemia, and obesity, can further amplify the prevalence of cardiovascular dysfunction following Hodgkin lymphoma treatment [23]. Notably, one study indicated that Hodgkin lymphoma patients with preexisting cardiac conditions face a particularly high risk of cardiac morbidity necessitating hospitalization following mediastinal irradiation [24].

The special linkage between other cancer types and TRAEs (as shown in Fig. 4A, B, and 4C), including hypothyroidism in gastric and gastroesophageal cancer, rash in prostate cancer, and neurological disorders in melanoma, makes it difficult to explain the possible underlying mechanisms. It has been hypothesized that immune-mediated destruction may unleash the presentation of previously unobserved self-antigens. These antigens can be found in tissues that are distant from the tumourigenic organ. Our current focus lies in offering support to patients with these cancers, including educating patients before treatment and performing routine tests before and during ICI therapy.

### 4.3 Comparison with other studies

Few previous systematic reviews and meta-analyses have quantified the AEs from ICIs across different cancer patients. A meta-analysis involving 20,128 patients elucidated the frequencies of common TRAEs associated with anti-PD-1 and anti-PD-L1 therapy, as well as the comparative TRAE rates across various drug classes. However, it did not delve into the examination of whether particular adverse events were more prevalent in specific cancer types, a gap that our study aimed to fill [25].

Khoja and colleagues systematically reviewed the distribution of immune-related AEs (IRAEs) of ICIs based on tumour type and ICI class [26]. It is recognized that IRAEs are classified and documented as a subset of TRAEs, which arise from immune responses affecting nontumour tissues in various organs [27]. Their findings revealed that all-grade colitis (OR 8.7, 95 % CI 5.8–12.9), hypophysitis (OR 6.5, 95 % CI 3.0–14.3) and rash (OR 2.0, 95 % CI 1.8–2.3) were more prevalent with CTLA-4 monoclonal antibodies. However, pneumonitis (OR 6.4, 95 % CI 3.2–12.7), hypothyroidism (OR 4.3, 95 % CI 2.9–6.3), arthralgia (OR 3.5, 95 % CI 2.6–4.8) and vitiligo (OR 3.5, 95 % CI 2.3–5.3) were more frequently observed with PD-1 inhibitors. We can see that these results are consistent with those presented in Supplemental material 2. Furthermore, they noted that patients with melanoma exhibited a higher incidence of gastrointestinal and cutaneous IRAEs, along with a lower prevalence of pneumonitis, which is consistent with our findings (Fig. 1). However, ambiguity still persists in the definition of IRAEs, as the assessment of whether an adverse effect is immune-related can be subjective, which might be susceptible to bias [28].

In a 2018 network meta-analysis, the comparative safety of eight treatment options was assessed, including five ICI monotherapies, one combination of ICI with conventional therapy, one conventional therapy, and one combination of two ICIs. Different doses of the same ICI drug were also explored across various cancers. The analysis identified atezolizumab as having the best overall safety profile, while nivolumab emerged as the safest option for lung cancer when considering a holistic perspective. In a dose-based network meta-analysis, they found no difference in the safety profile of each drug at different doses, except for ipilimumab [29]. In light of this finding, we conducted a subgroup comparison of the distribution of TRAEs associated with different doses of ipilimumab (10 mg per kg of body weight every 3 weeks vs. 3 mg per kg of body weight every 3 weeks) in melanoma patients (Fig. S17). The results revealed that the dose of ipilimumab at 3 mg/kg of body weight every 3 weeks is a safer choice than 10 mg/kg (RR 1.32, 95 % CI 1.25–1.40, P < 0.0001). Such findings can also serve as a valuable reference for determining the appropriate dosage when considering treatment with ipilimumab. Moreover, we also investigated the incidence of AEs associated with drugs targeting three different immune checkpoints across various follow-up time frames. Interestingly, we observed that not all outcomes exhibited a consistent increase with longer follow-up periods (Table S4). For instance, PD-1 inhibitors demonstrated the highest number of TRAEs after a follow-up period of more than 30 months, while the peak in the number of TRAEs between PD-L1 inhibitors and CTLA-4 inhibitors occurred within the 15- to 30-month follow-up range.

Additionally, we conducted a comparison of the overall safety of the three classes of ICIs in patients with metastatic and nonmetastatic malignancies (Table S5). Our analysis revealed a higher overall incidence of TRAEs in nonmetastatic cancer patients who received anti-PD-1/PD-L1 monotherapy. This elevated incidence can be attributed to the choice of ICI treatment by patients with resectable cancer before or after surgery. Based on these findings, we further explored whether a patient's treatment history influences the occurrence of ICI-induced adverse effects. Based on their treatment history, patients were divided into three groups: previously untreated, those with a history of surgery, and those with prior chemotherapy (Table S6). Interestingly, patients who had received prior surgery exhibited the highest overall incidence of TRAEs. Meanwhile, in patients with a history of prior chemotherapy, PD-1 inhibitors appeared to be the safest choice, whereas in previously untreated patients, PD-L1 inhibitors demonstrated the lowest overall incidence of TRAEs.

### 4.4 Strengths and limitations

Our meta-analysis includes several strengths. First, it represents the first study to systematically compare the occurrence of AEs associated with ICI monotherapy across different cancer types. Second, our rigorous search process and meticulous selection strategy for eligible trials lay the foundation for the quality and robustness of our study. Third, this analysis included many participants (26,277 participants) covering 16 cancer types and three kinds of ICI monotherapy. Fourth, among patients with the same cancer type, we further conducted subgroup comparisons of TRAE profiles between those who received ICIs in adjuvant settings and those in nonadjuvant contexts. In addition, we offer a more comprehensive distribution of TRAE details by organ system, accompanied by a range of treatment or prevention options offered by prior studies, which clinicians and patients can access in supplemental material 2. These data provide comprehensive evidence on the association of several TRAEs and specific cancer types.

However, this study has numerous shortcomings. First, we calculated study heterogeneity by calculating the ratio of patients who reported any TRAEs to all participants in each cancer type population. Nonetheless, not all RCTs supply data on the count of individuals experiencing at least one TRAE. Moreover, in certain cancer types, we encountered situations where only one study was available, which cannot be used in statistical tests for heterogeneity. This limitation could account for the I^2^ value exceeding 50 % in certain cancer types. Moreover, heterogeneity among RCTs may also stem from variations in inclusion criteria, patients' physical conditions (eg. metastatic or not), common comparator (e.g., chemotherapy), follow-up durations, and the history of prior failed treatment regimens. Therefore, we added subgroup analysis based on patient drug selection, treatment history, metastasis, etc. in Fig. S14. Second, current TRAE recording and reporting schemes are simple dichotomous scenarios with all-grade encompassing CTCAE 1-5 and serious-grade limited to CTCAE 3-5. However, when we attempted to investigate the distribution and severity of TRAEs with a more detailed and rigorous five-grade classification, the results did not meet our expectations. Third, adverse effects are often underreported on websites, typically limited to AEs with frequencies of at least 5 % or 10 %. Such incomplete reporting may introduce publication bias. While we strived for the utmost comprehensiveness and detail in collecting TRAE data from most RCTs by following the protocol involving searches on clinicaltrials.gov, examining supplemental materials, and comparing different literature records, we found it is nowhere near as detailed as treatment emergent AEs presented on clinicaltrials.gov. We strongly encourage future researchers to consider sharing comprehensive TRAE data, as this would significantly benefit subsequent learning and follow-up research endeavors.

### 4.5 Implications for clinical practice and policy

Over the past decade, we have witnessed the rapid development of ICI drugs while being plagued by their unpredictable toxicity, which can influence any organ system [11,30]. In this regard, our results offer valuable evidence to support the development of early prevention and personalized management strategies for ICI-related adverse effects.

Decision-makers can modify their treatment approaches based on our findings to reduce the incidence of TRAEs. For instance, patients with lung cancer receiving anti-PD-1 monotherapy demonstrated a higher likelihood of experiencing all-grade TRAEs than those receiving anti-PD-L1 therapy (see Fig. 4). However, renal cell carcinoma patients may find anti-PD-1 therapy a safer choice over PD-L1 inhibitors based on the risk ratio of overall TRAEs being 0.45 for anti-PD-L1 versus anti-PD-1 (95 % CI 0.42 to 0.48) (Fig. S8). When comparing TRAEs associated with anti-PD-1 monotherapy and anti-CTLA-4 monotherapy in melanoma patients, it becomes evident that the overall incidence of all-grade adverse effects is higher among anti-PD-1 patients. This observation suggests that, from a safety perspective, anti-PD-1 therapy may be the preferred choice (refer to Fig. S10). Consequently, our findings provide a relatively clear choice regarding selecting an ICI class in drug safety.

Furthermore, the overall incidence of TRAEs was higher among patients who received ICI therapy before or after surgery compared to those undergoing nonadjuvant treatment (Table S6). Sari Pesonen et al. proposed that this difference might be attributed to the heightened immunosuppressive state seen in patients with advanced cancer [31]. More importantly, regular monitoring and patient education are important for the early recognition and quick intervention of TRAEs. We recommend establishing a patient support service system on mobile technologies that can offer guidance on TRAE management, facilitate prompt reporting of patient conditions, and provide emotional support.

## 5 Conclusions

AEs related to ICI therapy among cancer patients are prevalent, and they lead to considerable costs for the health-care system. In this meta-analysis, we examined the distribution of AEs associated with ICI single-agent therapies across different cancer types. This comprehensive investigation aimed to provide insights into the question posed by Wang et al. in 2019: Are certain AEs more prevalent in specific types of cancer? [25] We found that within the same treatment group, the incidence rates of some TRAEs were higher in specific cancer types. Therefore, patients with different cancer types (including cancers of different histologic types) need to be aware of possible TRAEs when choosing ICI treatment modalities. It is also important to note that there is a significant difference between AEs induced by anti-PD-1 therapy and AEs induced by anti-PD-L1 therapy. Our results highlight the importance of considering potential AEs for choosing the best course of ICI treatment and building a convenient and reliable system of patient support services.

### 5.1 What is already known on this topic?

ICIs are commonly used to treat malignant tumors; however, they cause a wide range of TRAEs.

The profile and severity of TRAEs can vary between patients, even among patients using the same drug.

Previous results have suggested a positive correlation between the development of nonlethal immune-related TRAEs and the response to ICIs.

### 5.2 What this study adds

Under the same treatment regimen, the risk of TRAEs differed between cancer types.

Significant differences were found in the incidence of AEs induced by anti-PD-1 therapy versus AEs induced by anti-PD-L1 therapy in cancer patients.

When ICIs were administered as adjuvant therapy, the overall incidence of TRAEs was higher than when ICIs were administered as nonadjuvant therapy, and the distributions and incidence rates of TRAEs in adjuvant versus nonadjuvant settings were organ specific.

## CRediT authorship contribution statement

**Xuhui Tong:** Writing – review & editing, Writing – original draft, Visualization, Formal analysis. **Rong Tang:** Writing – review & editing, Writing – original draft. **Jin Xu:** Validation, Supervision, Methodology. **Wei Wang:** Validation, Supervision, Methodology. **Qiong Du:** Validation, Supervision, Methodology. **Si Shi:** Validation, Supervision, Methodology. **Xianjun Yu:** Supervision, Project administration, Methodology.

## Ethical approval

Not applicable.

## Data availability statement

No additional data are available.

## Funding

This study was jointly supported by the 10.13039/501100001809National Natural Science Foundation of China (U21A20374), Shanghai Municipal Science and Technology Major Project (21JC1401500), Scientific Innovation Project of Shanghai Education Committee (2019-01-07-00-07-E00057), and Natural Science Foundation of Shanghai (23ZR1479300). The funders had no role in considering the study design or in the collection, analysis, interpretation of data, writing of the report, or decision to submit the article for publication.

## Declaration of competing interest

The authors declare that there are no conflicts of interest regarding the publication of this manuscript, “Cancer type-specific adverse events of immune checkpoint inhibitors: A systematic review and meta-analysis.” The research was conducted in the absence of any commercial or financial relationships that could be construed as a potential conflict of interest.
