# Supplementary material for: Cancer type-specific adverse events of immune checkpoint inhibitors: A systematic review and meta-analysis
Source: Heliyon. 2025 Jan 2;11(1):e41597. doi: 10.1016/j.heliyon.2024.e41597 (PMC11757769; doi:10.1016/j.heliyon.2024.e41597)
Supplement: Multimedia component 4 [file mmc4.docx]

**Cancer type-specific adverse events of immune checkpoint inhibitors: A systematic review and meta-analysis**

Xuhui Tong, Rong Tang, Jin Xu, Wei Wang, Qiong Du, Si Shi, Xianjun Yu

**Supplemental material 1**

Table S1. Searching strategy

Table S2. Assessment of Risk of Bias Using the Cochrane Tool: Methodology and Criteria

Table S3. Baseline characteristics of patients included in meta-analysis.

Table S4. Quality assessment of randomized controlled trials using Cochrane risk of bias tool.

Table S5. Overall incidences of TRAEs of ICIs in different follow-up duration.

Table S6. Overall incidences of TRAEs of ICIs in patients with not metastatic vs metastatic cancer patients.

Table S7. Overall incidences of TRAEs of ICIs in patients previously untreated vs undergone previous surgery vs previous chemotherapy.

Table S8. Inconsistency Analysis of all grade TRAEs of included studies

Figure S1. Flow diagram for trial screen and selection.

Figure S2. Risk of bias of randomised controlled trials.

Figure S3. Funnel plots of included studies

Figure S4. Incidence rate (95% CI) of serious-grade TRAEs in cancer patients treated with single-agent anti-PD-1.

Figure S5. Incidence rate (95% CI) of serious-grade TRAEs in cancer patients treated with single-agent anti-PD-L1.

Figure S6. Incidence rate (95% CI) of serious-grade TRAEs in cancer patients treated with single-agent anti-CTLA-4.

Figure S7. Comparing TRAEs of lung cancer patients between anti-PD-1 and anti-PD-L1 treatment groups

Figure S8. Comparing TRAEs of renal cell carcinoma patients between anti-PD-1 and anti-PD-L1 treatment groups

Figure S9. Comparing TRAEs of urothelial carcinoma patients between anti-PD-1 and anti-PD-L1 treatment groups

Figure S10. Comparing TRAEs of melanoma patients between anti-PD-1 and anti-CTLA-4 treatment groups

Figure S11. Comparison of all-grade and serious TRAEs in NSCLC and SCLC with anti-PD-1 therapy.

Figure S12. Comparative safety of anti-PD-1 therapy in untreated versus previously ipilimumab treated melanoma patients.

Figure S13. Comparing TRAEs of lung cancer patients with single-agent anti-PD-1 as adjuvant and nonadjuvant therapy

Figure S14. Comparing TRAEs of melanoma patients with single-agent anti-PD-1 as adjuvant and nonadjuvant therapy

Figure S15. Comparing TRAEs of urothelial carcinoma patients with single-agent anti-PD-L1 as adjuvant and nonadjuvant therapy

Figure S16. Comparing TRAEs of melanoma patients with single-agent anti-CTLA-4 as adjuvant and nonadjuvant therapy

Figure S17. Comparing TRAEs of melanoma patients with single-agent anti-CTLA-4 at different doses.

Figure S18. Sensitivity analysis.

Figure S19. Subgroup analysis.

**Table S1. Searching strategy**

| Database | Keywords | Records |
| --- | --- | --- |
| **PubMed** | | **N= 2576** |
| #1 | ("Immune Checkpoint Inhibitors"[Mesh]) OR (PD-L1[Title/Abstract] OR anti PD-L1[Title/Abstract] OR Programmed Death-Ligand 1[Title/Abstract] OR anti Programmed Death-Ligand 1[Title/Abstract] OR PD-1-PD-L1 Blockade[Title/Abstract] OR CTLA-4[Title/Abstract] OR anti CTLA-4[Title/Abstract] OR Cytotoxic T-Lymphocyte-Associated Protein 4[Title/Abstract] OR anti Cytotoxic T-Lymphocyte-Associated Protein 4[Title/Abstract] OR PD-1[Title/Abstract] OR anti PD-1[Title/Abstract] OR Programmed Cell Death Protein 1[Title/Abstract] OR anti Programmed Cell Death Protein 1[Title/Abstract] OR CD40[Title/Abstract] OR anti CD40[Title/Abstract] OR CD40L[Title/Abstract] OR anti CD40L[Title/Abstract] OR CD154[Title/Abstract] OR anti CD154[Title/Abstract] OR LAG3[Title/Abstract] OR anti LAG3[Title/Abstract] OR Lymphocyte-activation gene 3[Title/Abstract] OR anti Lymphocyte-activation gene 3[Title/Abstract] OR Pembrolizumab[Title/Abstract] OR Balstilimab[Title/Abstract] OR Sintilimab[Title/Abstract] OR Nivolumab[Title/Abstract] OR Cemiplimab[Title/Abstract] OR Camrelizumab[Title/Abstract] OR Toripalimab[Title/Abstract] OR Tislelizumab[Title/Abstract] OR Spartalizumab[Title/Abstract] OR Atezolizumab[Title/Abstract] OR Avelumab[Title/Abstract] OR Durvalumab[Title/Abstract] OR Sugemalimab[Title/Abstract] OR SHR-1701[Title/Abstract] OR BMS202[Title/Abstract] OR BMS-936559[Title/Abstract] OR LY3300054[Title/Abstract] OR Iscalimab[Title/Abstract] OR BI 655064[Title/Abstract] OR Dacetuzumab[Title/Abstract] OR Lucatumumab[Title/Abstract] OR toralizumab[Title/Abstract] OR ipilimumab[Title/Abstract] OR Tremelimumab[Title/Abstract] OR Relatlimab[Title/Abstract] OR GSK2831781[Title/Abstract]) |  |
| #2 | ((((((cancer[Title/Abstract]) OR (carcinoma[Title/Abstract])) OR (adenocarcinoma[Title/Abstract])) OR (tumor[Title/Abstract])) OR (tumour[Title/Abstract])) OR (malignant[Title/Abstract])) OR (neoplasm[Title/Abstract]) |  |
| #3 | (clinical trial as topic[MeSH Terms]) OR (clinical trial[Publication Type]) |  |
| #4 | ("2012/01/01"[Date - Publication] : "2022/12/31"[Date - Publication]) |  |
| #5 | #1 AND #2 AND #3 AND #4 |  |
| #6 | ((((Review Literature as Topic[MeSH Terms]) OR (review[Publication Type]) OR (meta[Title] OR meta-analysis[Title] OR protocol[Title])) OR (comment[Publication Type])) OR (letter[Publication Type])) OR (meta-analysis[Publication Type]) |  |
| #7 | #5 NOT #6 |  |
| **The Cochrane Library** | |  |
| #1 | MeSH descriptor: [Immune Checkpoint Inhibitors] explode all trees | **N= 2073** |
| #2 | (PD-L1 OR PD-1 OR CTLA-4 OR CD40 OR CD40L OR CD154 OR LAG3 OR Pembrolizumab OR Balstilimab OR Sintilimab OR Nivolumab OR Cemiplimab OR Camrelizumab OR Toripalimab OR Tislelizumab OR Spartalizumab OR Atezolizumab OR Avelumab OR Durvalumab OR Sugemalimab OR SHR-1701 OR BMS202 OR BMS-936559 OR LY3300054 OR Iscalimab OR BI 655064 OR Dacetuzumab OR Lucatumumab OR toralizumab OR ipilimumab OR Tremelimumab OR Relatlimab OR GSK2831781):ti,ab,kw with Publication Year from 2012 to 2022, with Cochrane Library publication date Between Jan 2012 and Dec 2022, in Trials (Word variations have been searched) |  |
| #3 | (cancer OR carcinoma OR adenocarcinoma OR tumor OR tumour OR malignant OR neoplasm):ti,ab,kw with Publication Year from 2012 to 2022, with Cochrane Library publication date Between Jan 2012 and Dec 2022, in Trials (Word variations have been searched) |  |
| #4 | (clinical trials as topic or clinical trials):ti,ab,kw with Publication Year from 2012 to 2022, with Cochrane Library publication date Between Jan 2012 and Dec 2022, in Trials |  |
| #5 | #1 OR #2 |  |
| #6 | #5 AND #3 AND #4 |  |
| #7 | (conference OR review OR comment OR letter OR meta-analysis):pt with Publication Year from 2012 to 2022, with Cochrane Library publication date Between Jan 2012 and Dec 2022, in Trials (Word variations have been searched) |  |
| #8 | #6 NOT #7 |  |
| #9 | English:la with Publication Year from 2012 to 2022, with Cochrane Library publication date Between Jan 2012 and Dec 2022, in Trials (Word variations have been searched) |  |
| #10 | #8 AND #9 |  |
| **Web of Science** | | **N= 113** |
| #1 | ((TS=(Immune Checkpoint Inhibitors) OR AB=(PD-L1 OR anti PD-L1 OR Programmed Death-Ligand 1 OR anti Programmed Death-Ligand 1 OR PD-1-PD-L1 Blockade OR CTLA-4 OR anti CTLA-4 OR Cytotoxic T-Lymphocyte-Associated Protein 4 OR anti Cytotoxic T-Lymphocyte-Associated Protein 4 OR PD-1 OR anti PD-1 OR Programmed Cell Death Protein 1 OR anti Programmed Cell Death Protein 1 OR CD40 OR anti CD40 OR CD40L OR anti CD40L OR CD154 OR anti CD154 OR LAG3 OR anti LAG3 OR Lymphocyte-activation gene 3 OR anti Lymphocyte-activation gene 3 OR Pembrolizumab OR Balstilimab OR Sintilimab OR Nivolumab OR Cemiplimab OR Camrelizumab OR Toripalimab OR Tislelizumab OR Spartalizumab OR Atezolizumab OR Avelumab OR Durvalumab OR Sugemalimab OR SHR-1701 OR BMS202 OR BMS-936559 OR LY3300054 OR Iscalimab OR BI 655064 OR Dacetuzumab OR Lucatumumab OR toralizumab OR ipilimumab OR Tremelimumab OR Relatlimab OR GSK2831781)) AND (TS=(Clinical Trials as Topic)) AND (AB=(cancer OR carcinoma OR adenocarcinoma OR tumor OR tumour OR malignant OR neoplasm))) NOT (TS=(Review Literature as Topic OR meta-analysis OR protocol OR comment OR letter OR conference abstract) OR TI=(Review OR meta OR meta-analysis OR protocol)) |  |
| **Embase** | | **N= 1336** |
| #1 | ('immune checkpoint inhibitors':ab,ti OR 'pd l1':ab,ti OR 'anti pd-l1':ab,ti OR 'programmed death-ligand 1':ab,ti OR 'anti programmed death-ligand 1':ab,ti OR 'pd-1-pd-l1 blockade':ab,ti OR 'ctla 4':ab,ti OR 'anti ctla-4':ab,ti OR 'cytotoxic t-lymphocyte-associated protein 4':ab,ti OR 'anti cytotoxic t-lymphocyte-associated protein 4':ab,ti OR 'pd 1':ab,ti OR 'anti pd-1':ab,ti OR 'programmed cell death protein 1':ab,ti OR 'anti programmed cell death protein 1':ab,ti OR cd40:ab,ti OR 'anti cd40':ab,ti OR cd40l:ab,ti OR 'anti cd40l':ab,ti OR cd154:ab,ti OR 'anti cd154':ab,ti OR lag3:ab,ti OR 'anti lag3':ab,ti OR 'lymphocyte-activation gene 3':ab,ti OR 'anti lymphocyte-activation gene 3':ab,ti OR pembrolizumab:ab,ti OR balstilimab:ab,ti OR sintilimab:ab,ti OR nivolumab:ab,ti OR cemiplimab:ab,ti OR camrelizumab:ab,ti OR toripalimab:ab,ti OR tislelizumab:ab,ti OR spartalizumab:ab,ti OR atezolizumab:ab,ti OR avelumab:ab,ti OR durvalumab:ab,ti OR sugemalimab:ab,ti OR 'shr 1701':ab,ti OR bms202:ab,ti OR 'bms 936559':ab,ti OR ly3300054:ab,ti OR iscalimab:ab,ti OR 'bi 655064':ab,ti OR dacetuzumab:ab,ti OR lucatumumab:ab,ti OR toralizumab:ab,ti OR ipilimumab:ab,ti OR tremelimumab:ab,ti OR relatlimab:ab,ti OR gsk2831781:ab,ti) AND [controlled clinical trial]/lim AND [2012-2022]/py |  |
| #2 | (cancer:ab,ti OR carcinoma:ab,ti OR adenocarcinoma:ab,ti OR tumor:ab,ti OR tumour:ab,ti OR malignant:ab,ti OR neoplasm:ab,ti) AND [controlled clinical trial]/lim AND [2012-2022]/py |  |
| #3 | english:la AND [controlled clinical trial]/lim AND [2012-2022]/py |  |
| #4 | ('review'/exp OR review OR 'meta analysis'/exp OR 'meta analysis' OR 'protocol'/exp OR protocol OR comment OR 'letter'/exp OR letter OR 'conference abstract'/exp OR 'conference abstract' OR (('conference'/exp OR conference) AND ('abstract'/exp OR abstract))) AND [controlled clinical trial]/lim AND [2012-2022]/py |  |
| #5 | #1 AND #2 AND #3 |  |
| #6 | #5 NOT #4 |  |

**Figure S1. Flow diagram for trial screen and selection.**

**Identification of studies via databases and registers**

**Identification of studies via other methods**

**Identification**

Records identified from:

Citation searching (n = 4)

Records removed before screening:

Duplicate records removed (n = 2239)

Records identified from 4 databases

(n = 6098)

**Screening**

Reports sought for retrieval

(n = 1005)

Records screened (n = 3863)

Records excluded (n = 2858)

Reports excluded:

Insufficient AE data

Not RCT

Study design

Duplicate cohorts

Other

(n = 377)

(n = 174)

(n = 172)

(n = 113)

(n = 94)

Reports assessed for eligibility

(n = 1004)

**Included**

Studies included in meta-analysis (n = 75)

Detailed selection process:

**Initial Inclusion/Exclusion Criteria:** Duplicate records (2239 removed).

**Screening Process:** Wrong study type (879 removed), insufficient participants (22 removed), adverse events not reported (149 removed), no ICI alone therapy(1732 removed), different disease(74 removed).

**Quality Assessment:** Duplicate cohorts(115 removed), less participants (32 removed), no ICI alone therapy(47 removed), no TRAE reported (298 removed), insufficient information (195 removed).

**Final Selection Criteria:** not RCT (245 removed).

**75** studies for **final analysis.**

**Table S2. Assessment of Risk of Bias Using the Cochrane Tool: Methodology and Criteria**

| **Bias Domain** | **Low Risk Criteria** | **High Risk Criteria** | **Unclear Risk Criteria** |
| --- | --- | --- | --- |
| Random Sequence Generation | Adequate randomization method described | No randomization or inadequate method | Unclear randomization method described |
| Allocation Concealment | Allocation sequence concealed from participants and researchers | Allocation sequence not concealed | Unclear concealment method |
| Blinding of Participants and Staff | Participants and personnel blinded to interventions | No blinding or inadequately blinded | Unclear blinding status |
| Blinding of Outcome Assessment | Outcome assessors blinded to intervention allocation | No blinding or inadequately blinded | Unclear blinding status for outcome assessors |
| Incomplete Outcome Data | No missing outcome data or balanced across groups | Missing outcome data, imbalanced between groups | Unclear handling of missing data |
| Selective Reporting | All prespecified outcomes reported | Not all prespecified outcomes reported | Unclear reporting status |
| Other Bias | No other sources of bias identified | Other bias present | Unclear presence of other biases |

**Table S3. Baseline characteristics of patients included in meta-analysis.**

|  | **Patient characteristic** | Patients  (N = 26,277) |
| --- | --- | --- |
|  | **ICI target type**  PD-1  PD-L1  CTLA-4 | 18854  4858  2565 |
|  | **Cancer types**  Breast cancer  Cervical carcinoma  Colorectal carcinoma  Esophageal or gastroesophageal junction cancer  Gastric or gastroesophageal cancer  Glioblastoma  Head and neck carcinoma  Hepatocellular carcinoma  Hodgkin lymphoma  Lung cancer  Melanoma  Mesothelioma  Ovarian cancer  Prostate cancer  Renal cell carcinoma  Urothelial carcinoma | 425  300  153  1538  1062  182  1135  1263  148  8470  6442  221  343  399  1284  2912 |
|  | **Gender (%)**  Male | 70.4% |
|  | **Race (n=19727) (%)**  American Indian or Alaska Native  Asian  Black or African American  White  Native Hawaiian or Other Pacific Islander  Other  Unknown | 71 (0.36%)  4851 (24.59%)  308 (1.56%)  13874 (70.33%)  27 (0.14%)  257 (1.3%)  339 (1.72%) |
|  | **Median follow-up (n=25467)**  < 15 months  15 months to 30 months  >30 months | 10631  6993  7843 |
|  | **Treatment history**  Chemotherapy  surgery  Untreated  Previous treatment not clarified | 7041  5397  4532  6414 |
|  | **Metastatic or not (n=16364)**  Metastatic  Not metastatic | 12157  207 |
|  | **PD-L1 expression (n=17485)**  ≥1%  <1% | 9588  4817 |

**Table S4. Quality assessment of randomized controlled trials using Cochrane risk of bias tool.**

| Study | Trial ID | Random sequence generation | Allocation concealment | Blinding of participants and personnel | Blinding of outcome assessment | Incomplete outcome data | Selective reporting | Other bias | Overall Risk |
| --- | --- | --- | --- | --- | --- | --- | --- | --- | --- |
| Joaquim 2021(1) | NCT02450331 | Low risk | Low risk | High risk | High risk | Low risk | Low risk | Low risk | High risk |
| Sumanta 2022(2) | NCT03024996 | Low risk | Low risk | Low risk | High risk | Low risk | Low risk | Low risk | High risk |
| Ronan 2021(3) | NCT02743494 | Low risk | Unclear | Low risk | High risk | Low risk | Low risk | Low risk | High risk |
| Jeffrey 2017(4) | NCT02388906 | Low risk | Low risk | Low risk | Low risk | Low risk | Low risk | Low risk | Low risk |
| Dean 2021(5) | NCT02632409 | Low risk | Low risk | Low risk | High risk | Low risk | Low risk | Unclear | High risk |
| Kenneth 2022(6) | NCT02506153 | Low risk | Low risk | Low risk | High risk | Low risk | Low risk | Low risk | High risk |
| Alexander 2018(7) | NCT02362594 | Low risk | Low risk | Low risk | High risk | Low risk | Low risk | Low risk | High risk |
| D 2020(8) | NCT02352948 | Low risk | Low risk | High risk | High risk | Low risk | Low risk | Low risk | High risk |
| Thomas 2018(9) | NCT02302807 | Low risk | Unclear | High risk | High risk | Low risk | Low risk | Low risk | High risk |
| Louis 2016(10) | NCT01903993 | Low risk | Low risk | High risk | High risk | Low risk | Low risk | Unclear | High risk |
| Matthew 2020(11) | NCT02807636 | Low risk | Low risk | Low risk | High risk | Low risk | Low risk | Low risk | High risk |
| Eric 2021(12) | NCT02580058 | Low risk | Low risk | High risk | High risk | Low risk | Low risk | Low risk | High risk |
| Thomas 2020(13) | NCT02603432 | Low risk | Unclear | High risk | High risk | Low risk | Low risk | Low risk | High risk |
| Fabrice 2018(14) | NCT02395172 | Low risk | Unclear | High risk | High risk | Low risk | Low risk | Low risk | High risk |
| Shukui 2020(15) | NCT02989922 | Low risk | Low risk | High risk | High risk | Low risk | Low risk | Low risk | High risk |
| Jing 2020(16) | NCT03099382 | Low risk | Low risk | High risk | High risk | Low risk | Low risk | Low risk | High risk |
| Ahmet 2021(17) | NCT03088540 | Low risk | Low risk | High risk | High risk | Low risk | Low risk | Low risk | High risk |
| Scott 2017(18) | NCT02125461 | Low risk | Low risk | Low risk | Low risk | Low risk | Low risk | Low risk | Low risk |
| Thomas 2020(19) | NCT02516241 | Low risk | Low risk | High risk | High risk | Low risk | Low risk | Low risk | High risk |
| R L Ferris 2020(20) | NCT02369874 | Low risk | Unclear | High risk | High risk | Low risk | Low risk | Low risk | High risk |
| Naiyer 2020(21) | NCT02453282 | Low risk | Low risk | High risk | High risk | Low risk | Low risk | Low risk | High risk |
| David 2020(22) | NCT02017717 | Low risk | Low risk | High risk | High risk | Low risk | Low risk | Low risk | High risk |
| Kohei 2020(23) | NCT02494583 | Low risk | Unclear | Low risk | Low risk | Low risk | Low risk | Low risk | Unclear |
| David 2017(24) | NCT02041533 | Low risk | Unclear | High risk | High risk | Low risk | Low risk | Low risk | High risk |
| Gilberto 2022(25) | NCT02220894 | Low risk | Unclear | High risk | High risk | Low risk | Low risk | Low risk | High risk |
| Martin 2021(26) | NCT02142738 | Low risk | Unclear | High risk | High risk | Low risk | Low risk | Unclear | High risk |
| Antoine 2022(27) | NCT02564263 | Low risk | Unclear | High risk | High risk | Low risk | Low risk | Low risk | High risk |
| Leisha 2019(28) | NCT01375842 | Low risk | Unclear | High risk | High risk | Low risk | Low risk | Unclear | High risk |
| Roy 2020(29) | NCT01905657 | Low risk | Low risk | High risk | High risk | Low risk | Low risk | Low risk | High risk |
| Jedd D 2022(30) | NCT01844505 | Low risk | Low risk | Low risk | Low risk | Low risk | Low risk | Low risk | Low risk |
| Taofeek 2021(31) | NCT02538666 | Low risk | Unclear | Low risk | Low risk | Low risk | Low risk | Unclear | Unclear |
| Robert 2016(32) | NCT02105636 | Low risk | Low risk | Low risk | High risk | Low risk | Low risk | Low risk | High risk |
| Yoon-Koo 2017(33) | NCT02267343 | Low risk | Unclear | Low risk | Low risk | Low risk | Low risk | Low risk | Unclear |
| Caroline 2015(34) | NCT01721772 | Low risk | Unclear | Low risk | Unclear | Low risk | Low risk | Low risk | Unclear |
| Jeffrey 2015(35) | NCT01721746 | Low risk | Low risk | High risk | High risk | Low risk | Low risk | Low risk | High risk |
| Ken 2019(36) | NCT02569242 | Low risk | Low risk | High risk | High risk | Low risk | Low risk | Low risk | High risk |
| Shun 2021(37) | NCT02613507 | Low risk | Unclear | High risk | High risk | Low risk | Low risk | Low risk | High risk |
| Hossein 2015(38) | NCT01673867 | Low risk | Unclear | High risk | High risk | Low risk | Low risk | Unclear | High risk |
| Julie 2015(39) | NCT01642004 | Low risk | Unclear | High risk | High risk | Low risk | Low risk | Low risk | High risk |
| Robert 2015(40) | NCT01668784 | Low risk | Low risk | High risk | High risk | Low risk | Low risk | Low risk | High risk |
| Junzo 2021(41) | JapicCTI-153004 | Low risk | Unclear | High risk | High risk | Low risk | Low risk | Unclear | High risk |
| Dean 2021(42) | NCT03063450 | Low risk | Low risk | High risk | High risk | Low risk | Low risk | Low risk | High risk |
| Thomas 2022(43) | NCT02576509 | Low risk | Low risk | High risk | High risk | Low risk | Low risk | Low risk | High risk |
| Paolo 2020(44) | NCT01515189 | Low risk | Low risk | Low risk | Low risk | Low risk | Low risk | Low risk | Low risk |
| Thomas 2021(45) | NCT02853305 | Low risk | Low risk | High risk | High risk | Low risk | Low risk | Low risk | High risk |
| Richard 2020(46) | NCT02256436 | Low risk | Low risk | Low risk | High risk | Low risk | Low risk | Low risk | High risk |
| Joaquim 2017(47) | NCT02702401 | Low risk | Unclear | High risk | High risk | Low risk | Low risk | Low risk | High risk |
| Edward 2015(48) | NCT01295827 | Low risk | High risk | High risk | High risk | Low risk | Low risk | Unclear | High risk |
| Thierry 2022(49) | NCT02563002 | Low risk | Low risk | High risk | High risk | Low risk | Low risk | Low risk | High risk |
| Arjun 2021(50) | NCT02625961 | Low risk | Unclear | High risk | High risk | Low risk | Low risk | Low risk | High risk |
| A2022(51) | NCT02611960 | Low risk | Low risk | Low risk | Low risk | Low risk | Low risk | Low risk | Low risk |
| John 2021(52) | NCT02684292 | Low risk | Low risk | High risk | High risk | Low risk | Low risk | Low risk | High risk |
| Antoni 2015(53) | NCT01704287 | Low risk | Low risk | Low risk | High risk | Low risk | Low risk | Low risk | High risk |
| Eric 2021(54) | NCT02555657 | Low risk | Low risk | High risk | High risk | Low risk | Low risk | Low risk | High risk |
| Caroline 2015(55) | NCT01866319 | Low risk | Low risk | High risk | High risk | Low risk | Low risk | Unclear | High risk |
| Ezra2019(56) | NCT02252042 | Low risk | Unclear | High risk | High risk | Low risk | Low risk | Unclear | High risk |
| Kohei 2018(57) | NCT02370498 | Low risk | Low risk | High risk | High risk | Low risk | Low risk | Unclear | High risk |
| Mary 2022(58) | NCT02504372 | Low risk | Low risk | Low risk | Low risk | Low risk | Low risk | Low risk | Low risk |
| Jason 2022(59) | NCT03553836 | Low risk | Low risk | Low risk | Low risk | Low risk | Low risk | Low risk | Low risk |
| Thomas 2022(60) | NCT03142334 | Low risk | Low risk | Low risk | Low risk | Low risk | Low risk | Low risk | Low risk |
| Shukui 2022(61) | NCT03062358 | Low risk | Low risk | Low risk | High risk | Low risk | Low risk | Low risk | High risk |
| Kevin 2022(62) | NCT02358031 | Low risk | Low risk | High risk | High risk | Low risk | Low risk | Low risk | High risk |
| Y-J Bang 2018(63) | NCT02625623 | Low risk | Unclear | High risk | High risk | Low risk | Low risk | Low risk | High risk |
| Yi-Long 2019(64) | NCT03850444 | Low risk | Unclear | High risk | High risk | Low risk | Low risk | Unclear | High risk |
| Tomasz 2017(65) | NCT01057810 | Low risk | Unclear | High risk | High risk | Low risk | Low risk | Low risk | High risk |
| Hussein 2022(66) | NCT03470922 | Low risk | Unclear | Low risk | Low risk | Low risk | Low risk | Low risk | Unclear |
| Omid 2013(67) | NCT01295827 | Low risk | Low risk | Unclear | Unclear | Low risk | Low risk | Low risk | Unclear |
| David 2020(68) | NCT02066636 | Low risk | Unclear | High risk | High risk | Low risk | Low risk | Low risk | High risk |
| Robin 2021(69) | NCT02519348 | Low risk | Unclear | High risk | High risk | Low risk | Low risk | Low risk | High risk |
| D R Spigel2021(70) | NCT02481830 | Low risk | Low risk | High risk | High risk | Low risk | Low risk | Low risk | High risk |
| Yuankai 2022(71) | NCT03150875 | Low risk | Low risk | High risk | High risk | Low risk | Low risk | Low risk | High risk |
| Qing 2022(72) | NCT03728556 | Low risk | Low risk | Low risk | Low risk | Low risk | Low risk | Low risk | Low risk |
| Krishnansu 2022(73) | NCT03257267 | Low risk | Low risk | High risk | High risk | Low risk | Low risk | Low risk | High risk |
| Lin 2022(74) | NCT03430843 | Low risk | Low risk | High risk | High risk | Low risk | Low risk | Low risk | High risk |
| Caicun 2022(75) | NCT03358875 | Low risk | Unclear | High risk | High risk | Low risk | Low risk | Low risk | High risk |
|  | | | | | | | | | |
| **Overall risk of bias (n=75）** | |  | | | | | | | |
| Low | | 75 | 46 | 24 | 13 | 75 | 75 | 63 | 9 |
| Unclear | | 0 | 28 | 1 | 2 | 0 | 0 | 12 | 6 |
| High | | 0 | 1 | 50 | 60 | 0 | 0 | 0 | 60 |

**Risk of bias assessment and publication bias**

Among the 75 RCTs, 60 trials exhibited at least one domain classified as having a high risk of bias, most commonly because of lack of blinding of participants and personnel (67%) or blinding outcome assessment (81%) (see Table S3 and Figure S2). In 50 trials, both participants and trial staff were not masked, resulting in a high risk of performance and detection bias. However, the modified funnel plots did not display any significant asymmetry, suggesting an absence of substantial publication bias. This observation was further confirmed by the results of Egger’s test (Figure S3).

**Figure S2. Risk of bias of randomized controlled trials.**


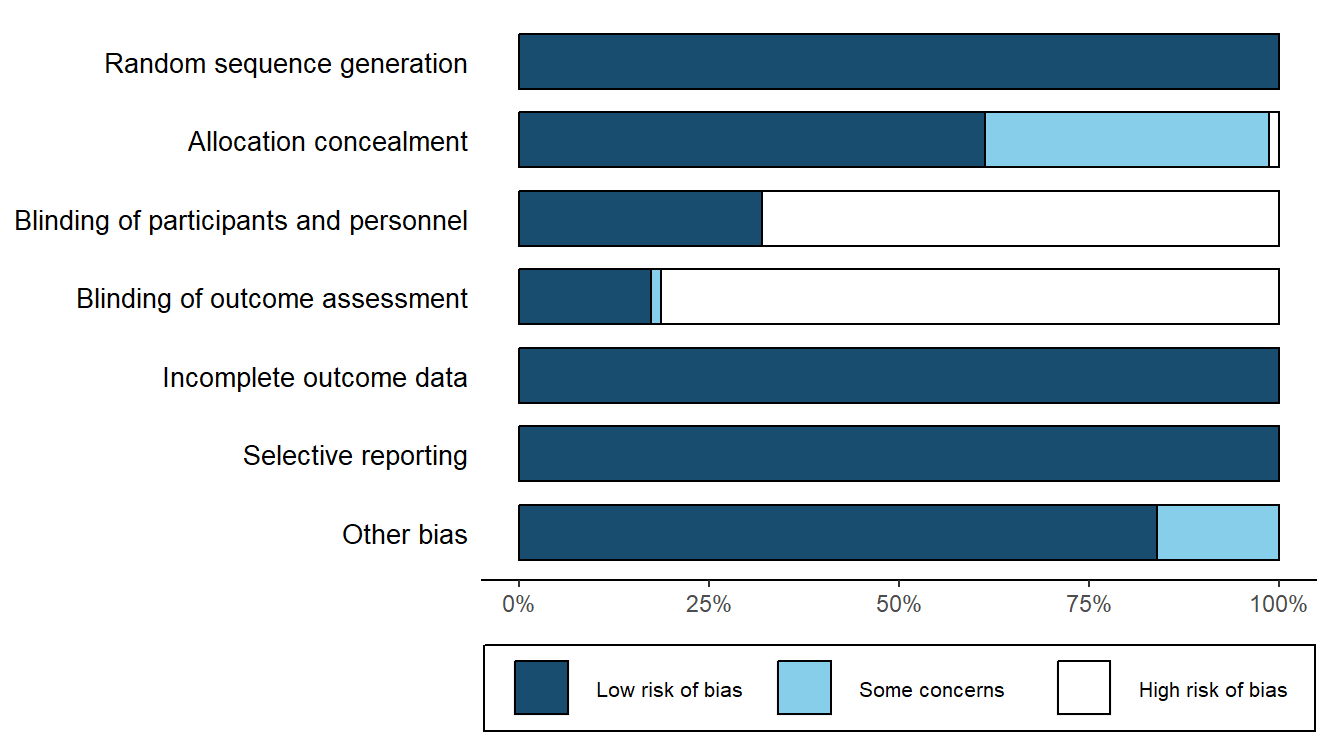


**Figure S3. Funnel plots of included studies**


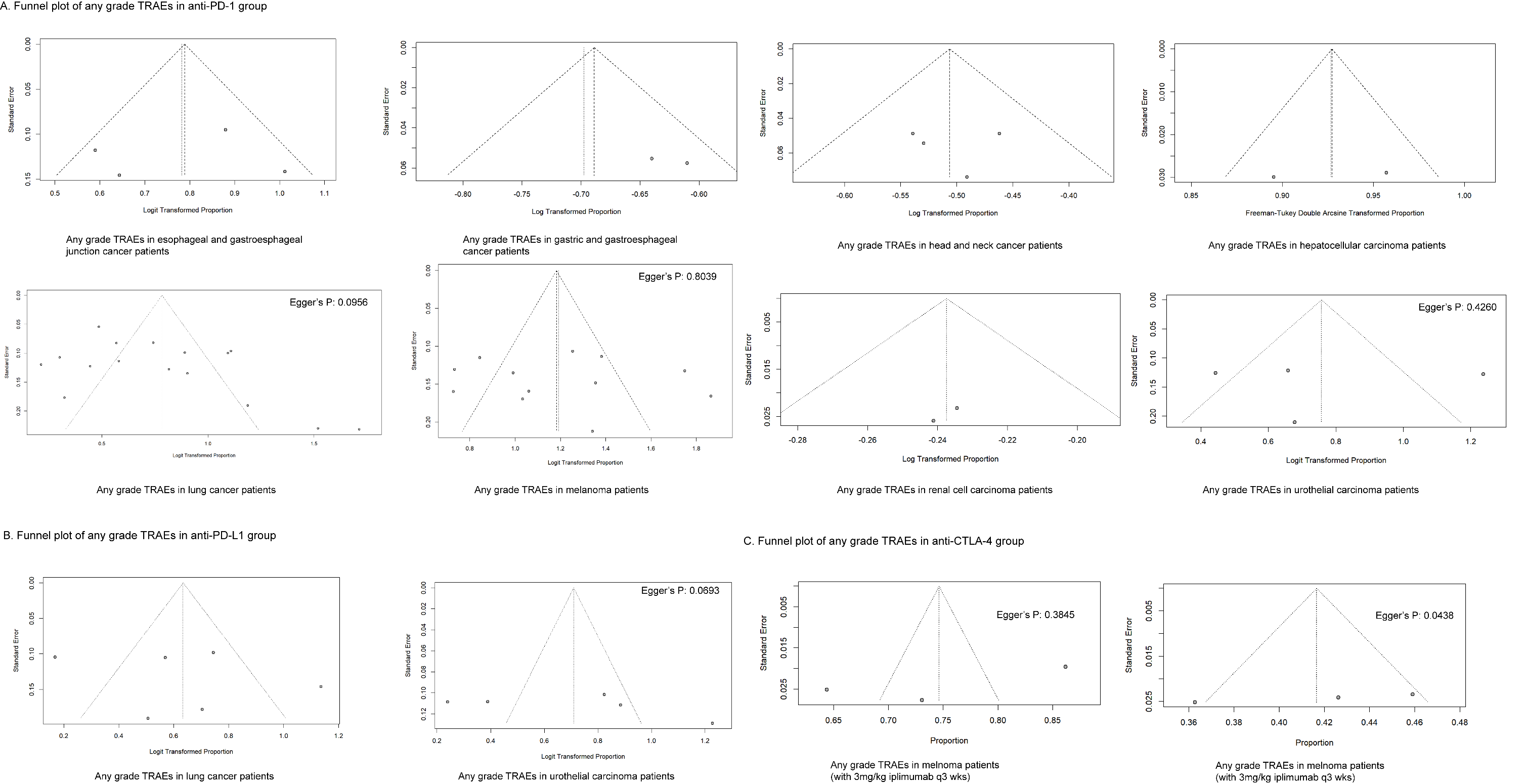


**Figure S4. Incidence rate (95% CI) of serious-grade TRAEs in cancer patients treated with single-agent anti-PD-1.**


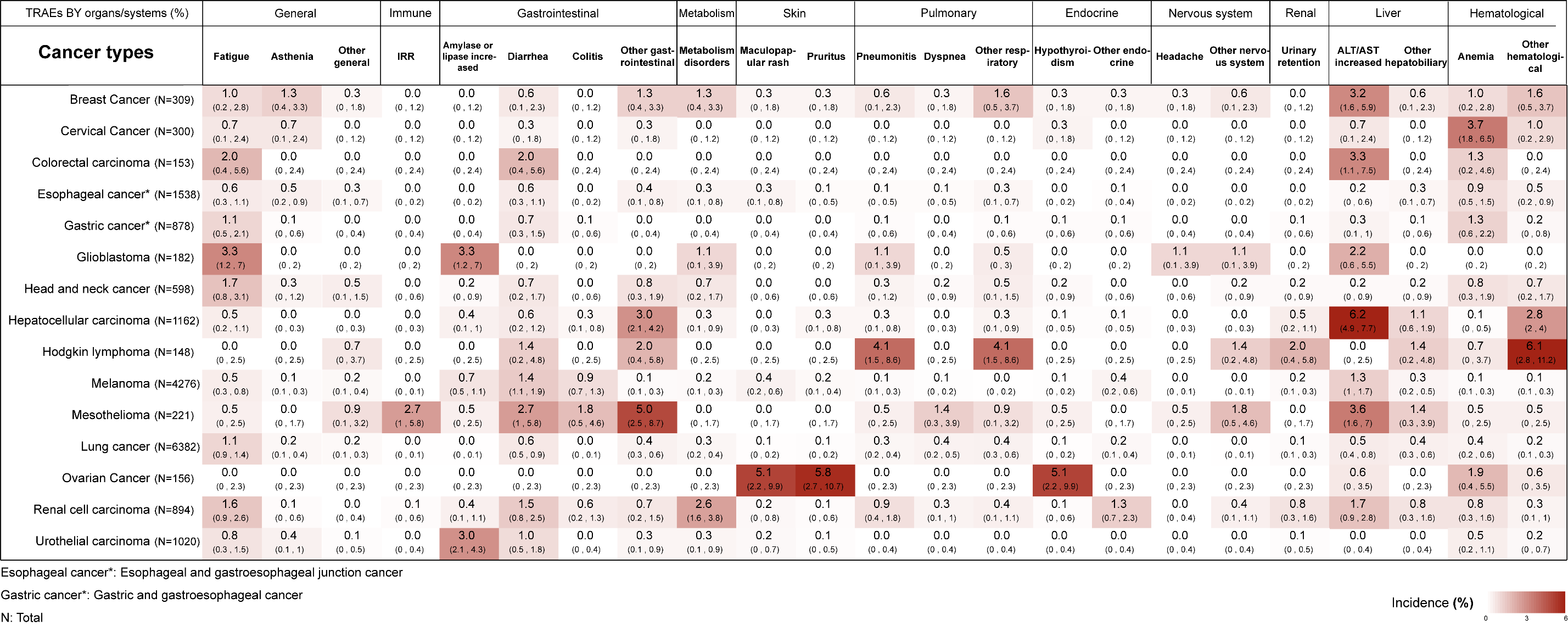


**Figure S5. Incidence rate (95% CI) of serious-grade TRAEs in cancer patients treated with single-agent anti-PD-L1.**


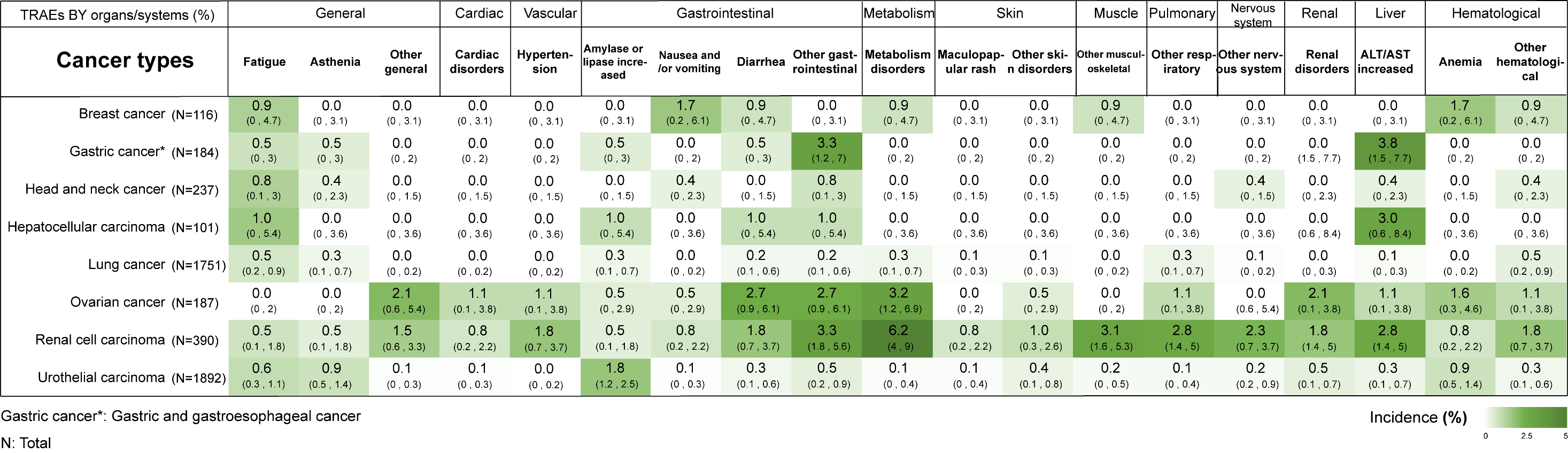


**Figure S6. Incidence rate (95% CI) of serious-grade TRAEs in cancer patients treated with single-agent anti-CTLA-4.**


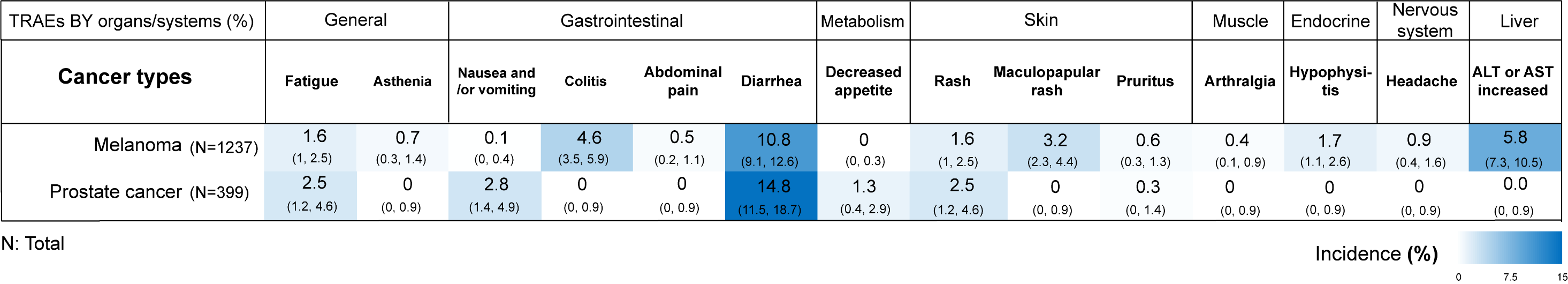


A total of 73 studies reported the distribution of serious TRAEs across different cancer types. The distribution and heatmaps of serious TRAEs across 16 kinds of malignancies in different ICI monotherapy groups are shown in Figures S4, S5, and S6.

In the anti-PD-1 monotherapy group, severe cutaneous disorders were more frequently seen in ovarian cancer than in other cancer types. For example, 5.8% (95% CI 2.7%-10.7%) and 5.1% (95% CI 2.2%-9.9%) of ovarian cancer patients experienced treatment-related pruritus and maculopapular rash, respectively. Severe treatment-associated fatigue was most frequently observed in glioblastoma patients (3.3%, 95% CI 1.2-7.0%), and a higher frequency of other serious gastrointestinal disorders occurred in patients with mesothelioma (5.0%, 95% CI 2.5%-8.7%). Furthermore, the incidences of both treatment-related pneumonitis and other severe respiratory system disorders were highest in Hodgkin lymphoma (4.1%, 95% CI 1.5%-8.6%; 4.1%, 95% CI 1.5%-8.6%, respectively). In addition, 5.1% of ovarian cancer patients experienced severe hypothyroidism (95% CI 2.2%-9.9%).

Among patients treated with PD-L1 monoclonal antibodies (Figure S5), individuals with renal cell carcinoma had the highest incidence of serious metabolic problems, other musculoskeletal issues, and other pulmonary disorders, with rates of 6.2% (95% CI 4.0%-9.0%), 3.1% (95% CI 1.6%-5.3%), and 2.8% (95% CI 1.4%-5.0%), respectively. A grade 3-5 ALT/AST increase was most frequently observed in patients with gastric and gastroesophageal cancer (3.8%, 95% CI 1.5%-7.7%). The highest proportion of severe diarrhoea and other gastrointestinal disorders occurred in ovarian cancer patients (2.7%, 95% CI 0.9%-6.1%; 2.7%, 95% CI 0.9%-6.1%, respectively).

We found that diarrhoea was the most frequent serious TRAE in both melanoma and prostate cancer patients. Fifty-nine out of 399 prostate cancer patients experienced severe diarrhoea (14.8%, 95% CI 11.5%-18.7%), and both severe fatigue and rash were observed in 2.5% of patients with prostate cancer (95% CI 1.2%-4.6%). A total of 5.8% of melanoma patients experienced abnormal ALT/AST levels (95% CI 7.3%-10.5%), and colitis occurred in 4.6% of melanoma patients (95% CI 35%-5.9%).

**Figure S7. Comparing TRAEs of lung cancer patients between anti-PD-1 and anti-PD-L1 treatment groups**


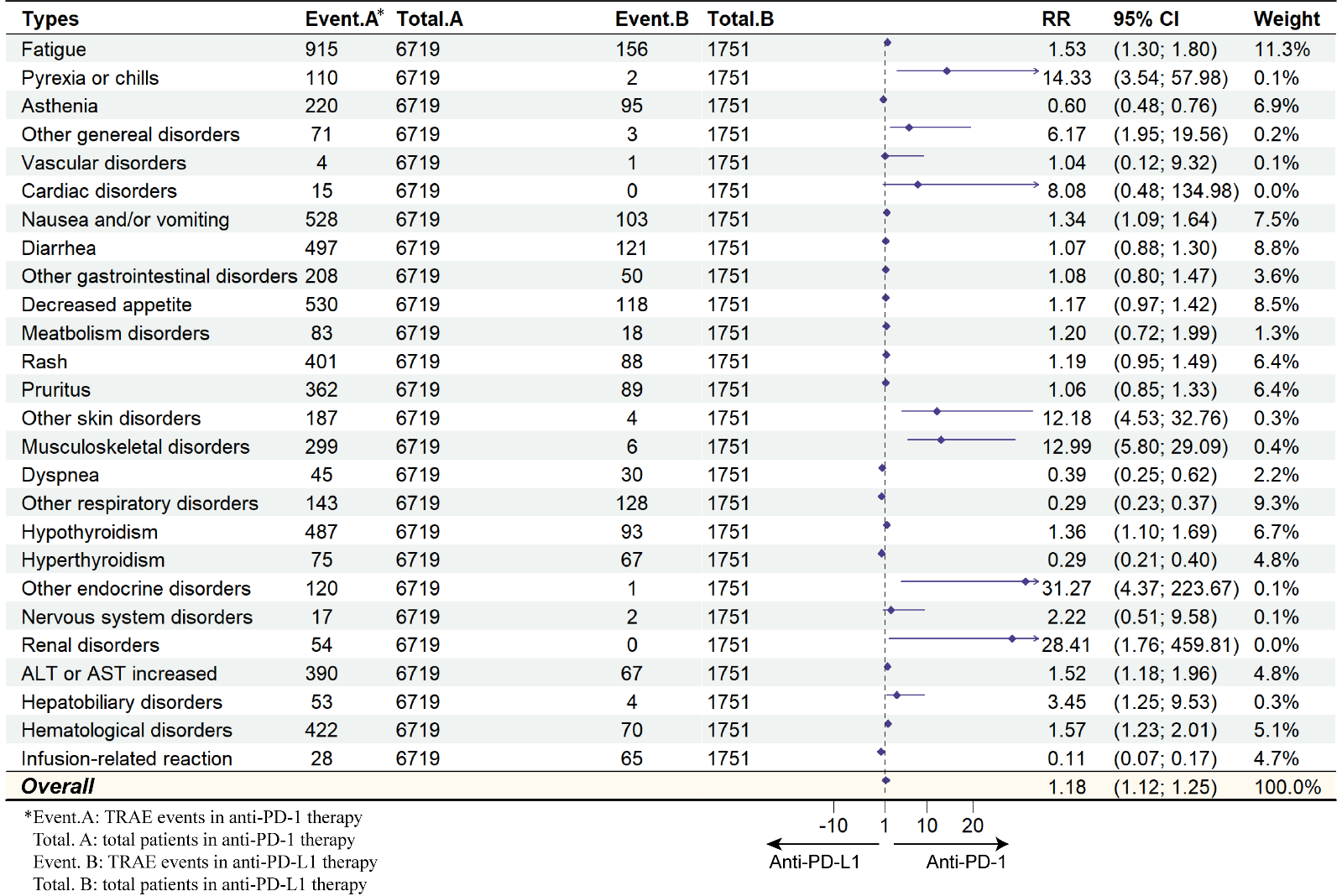


**Figure S8. Comparing TRAEs of renal cell carcinoma patients between anti-PD-1 and anti-PD-L1 treatment groups**


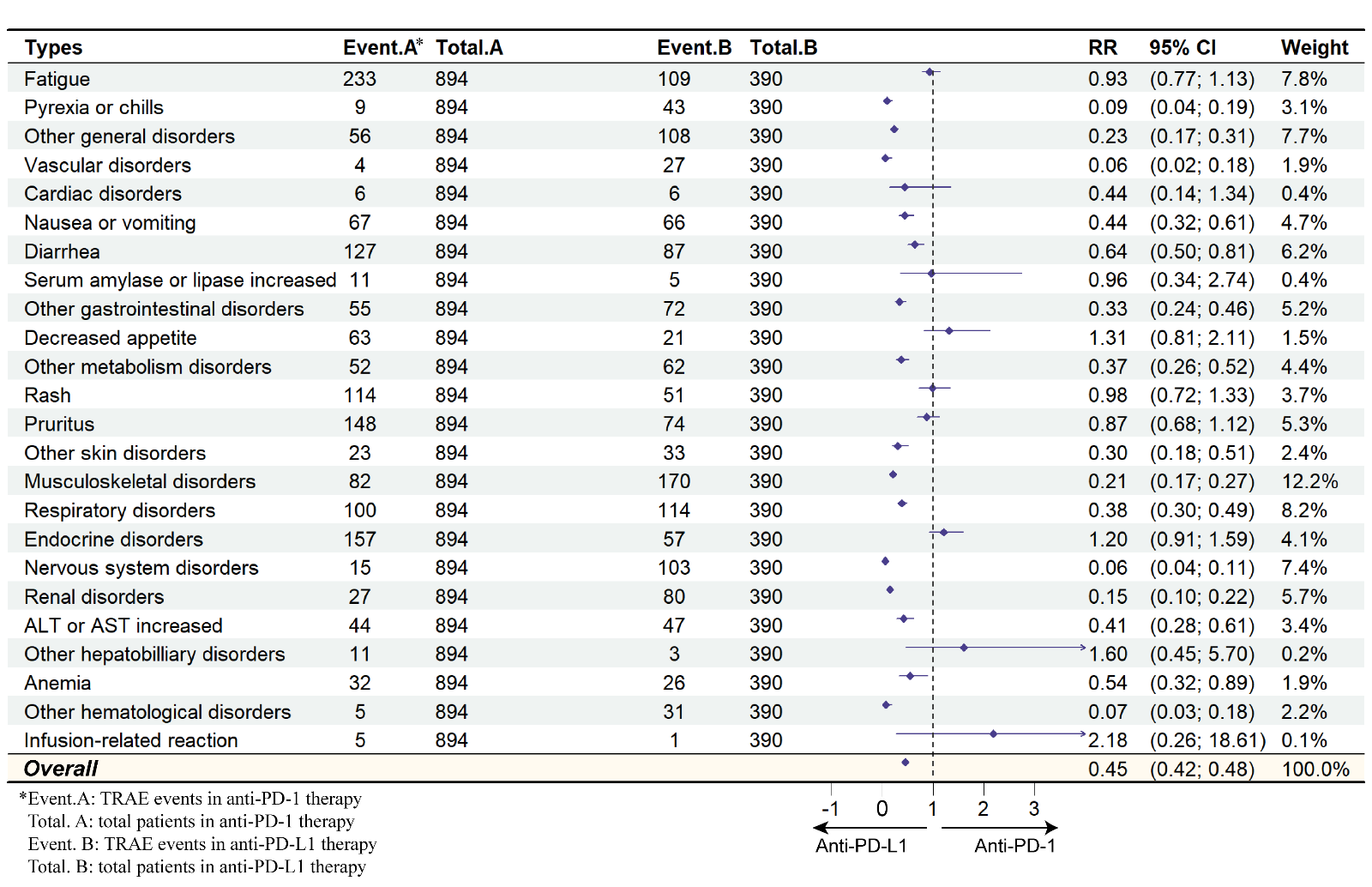


**Figure S9. Comparing TRAEs of urothelial carcinoma patients between anti-PD-1 and anti-PD-L1 treatment groups**


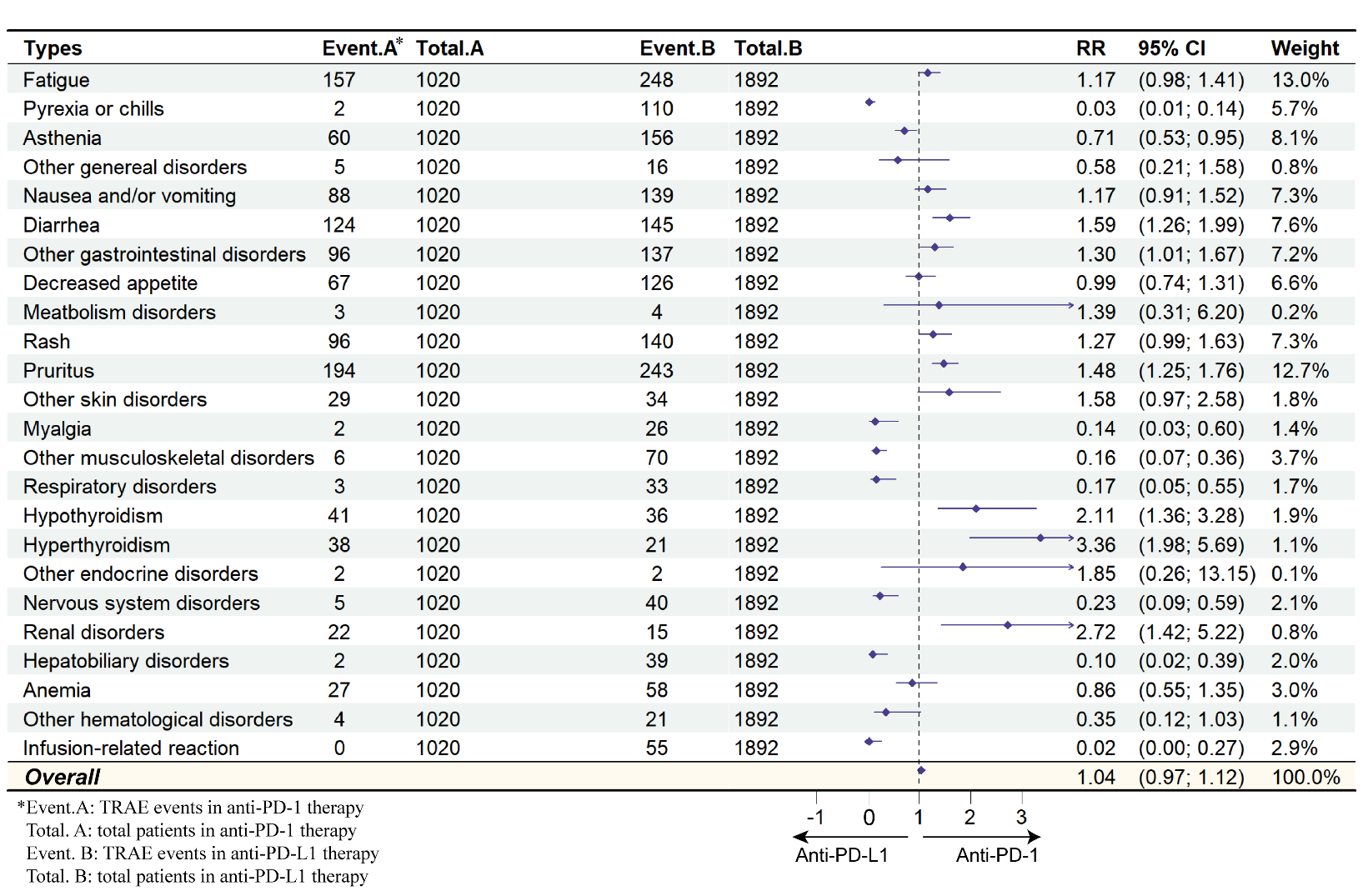


**Figure S10. Comparing TRAEs of melanoma patients between anti-PD-1 and anti-CTLA-4 treatment groups**


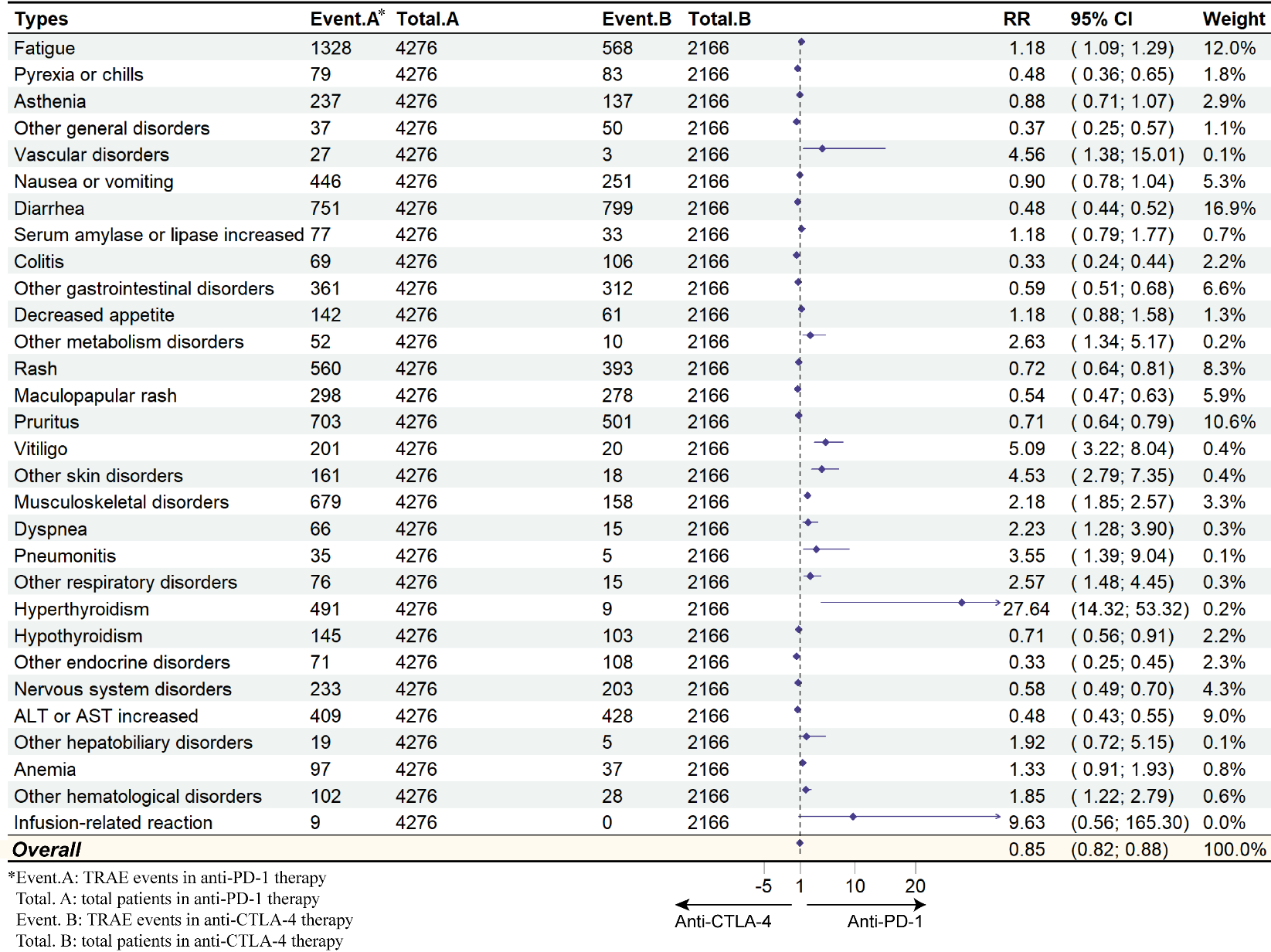


**Figure S11. Comparison of all-grade and serious TRAEs in NSCLC and SCLC with anti-PD-1 therapy.**


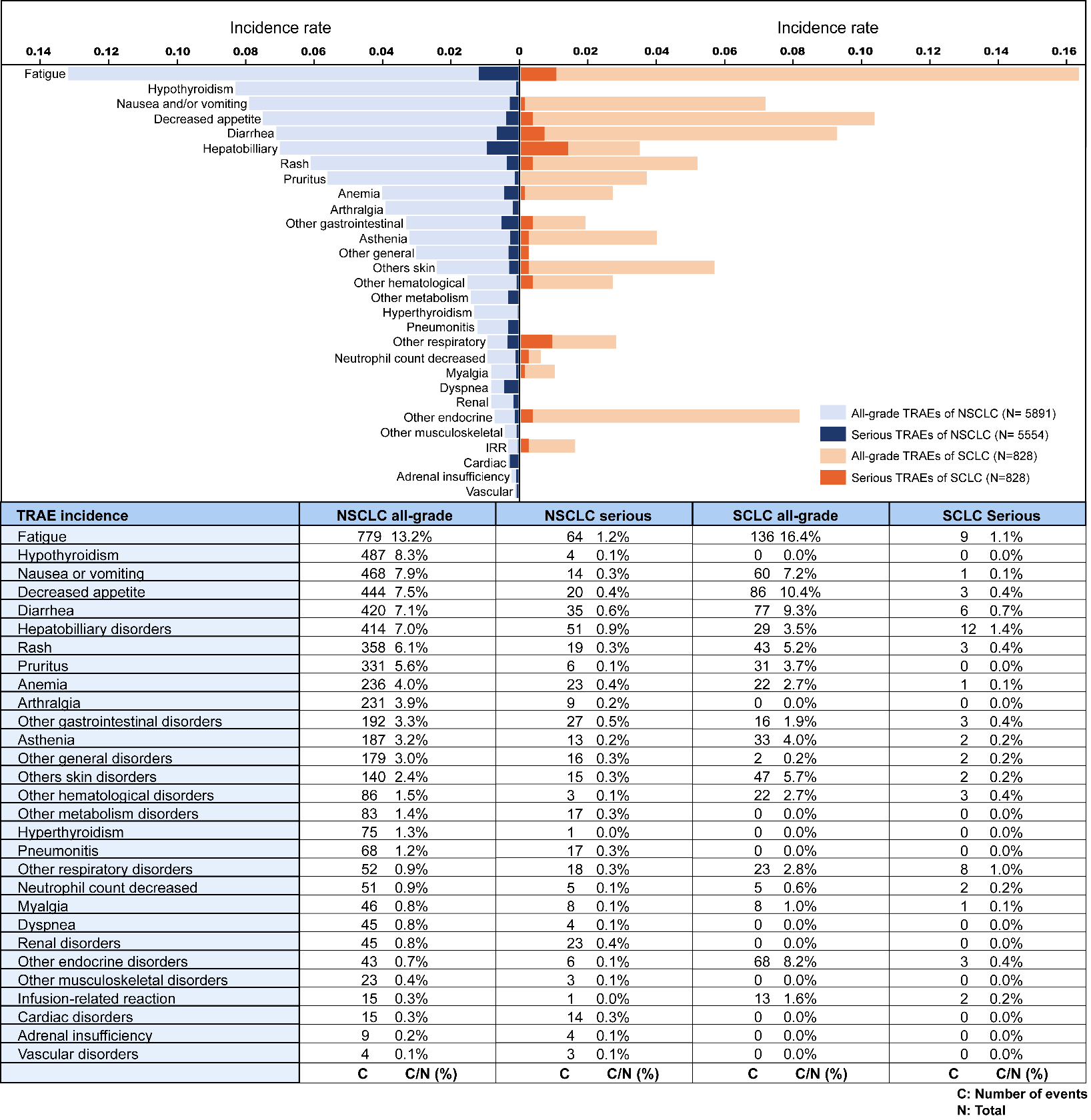


**Figure S12. Comparative safety of anti-PD-1 therapy in untreated versus previously ipilimumab treated melanoma patients.**


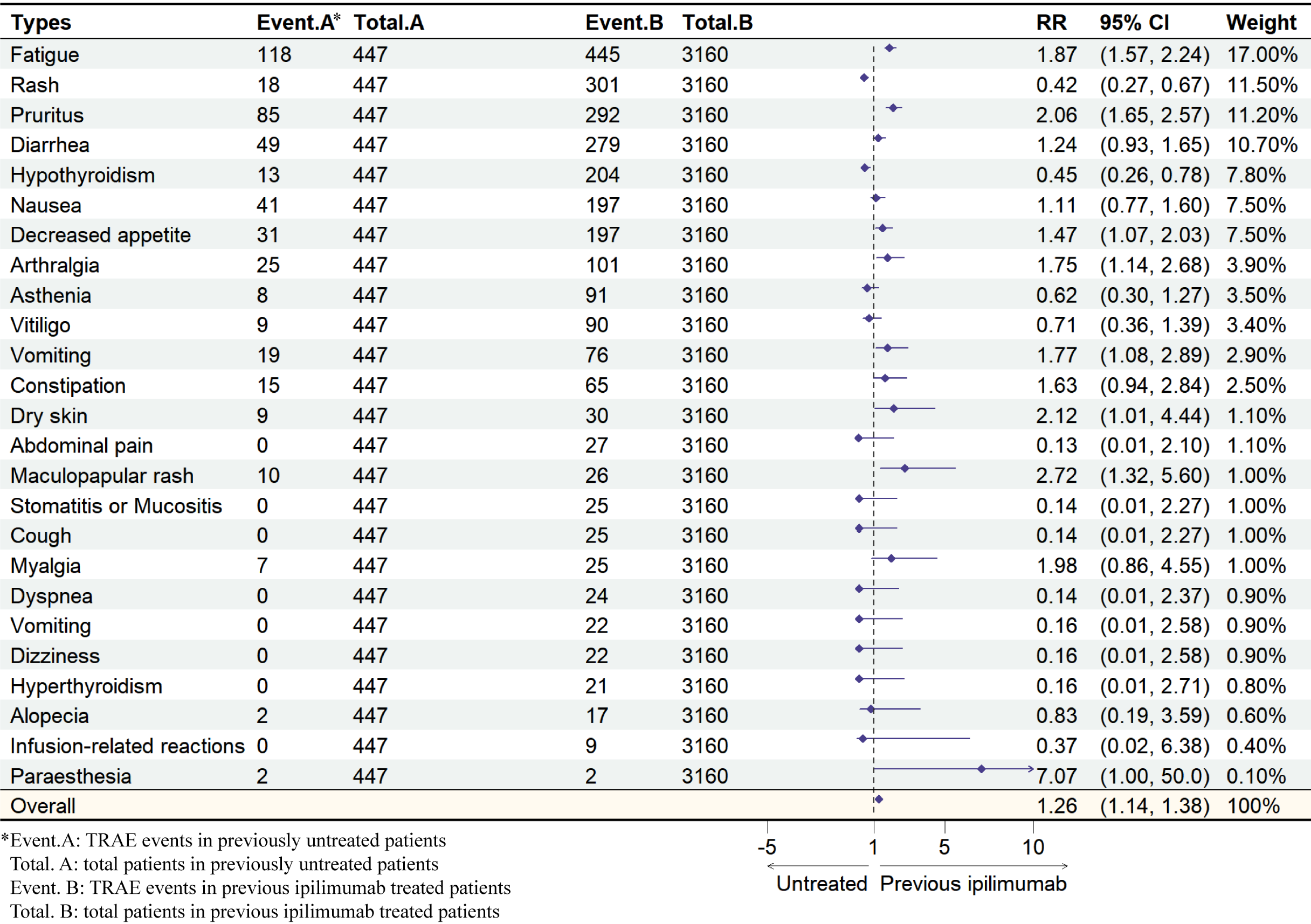


**Figure S13.** **Comparing TRAEs of lung cancer patients with single-agent anti-PD-1 as adjuvant and nonadjuvant therapy**


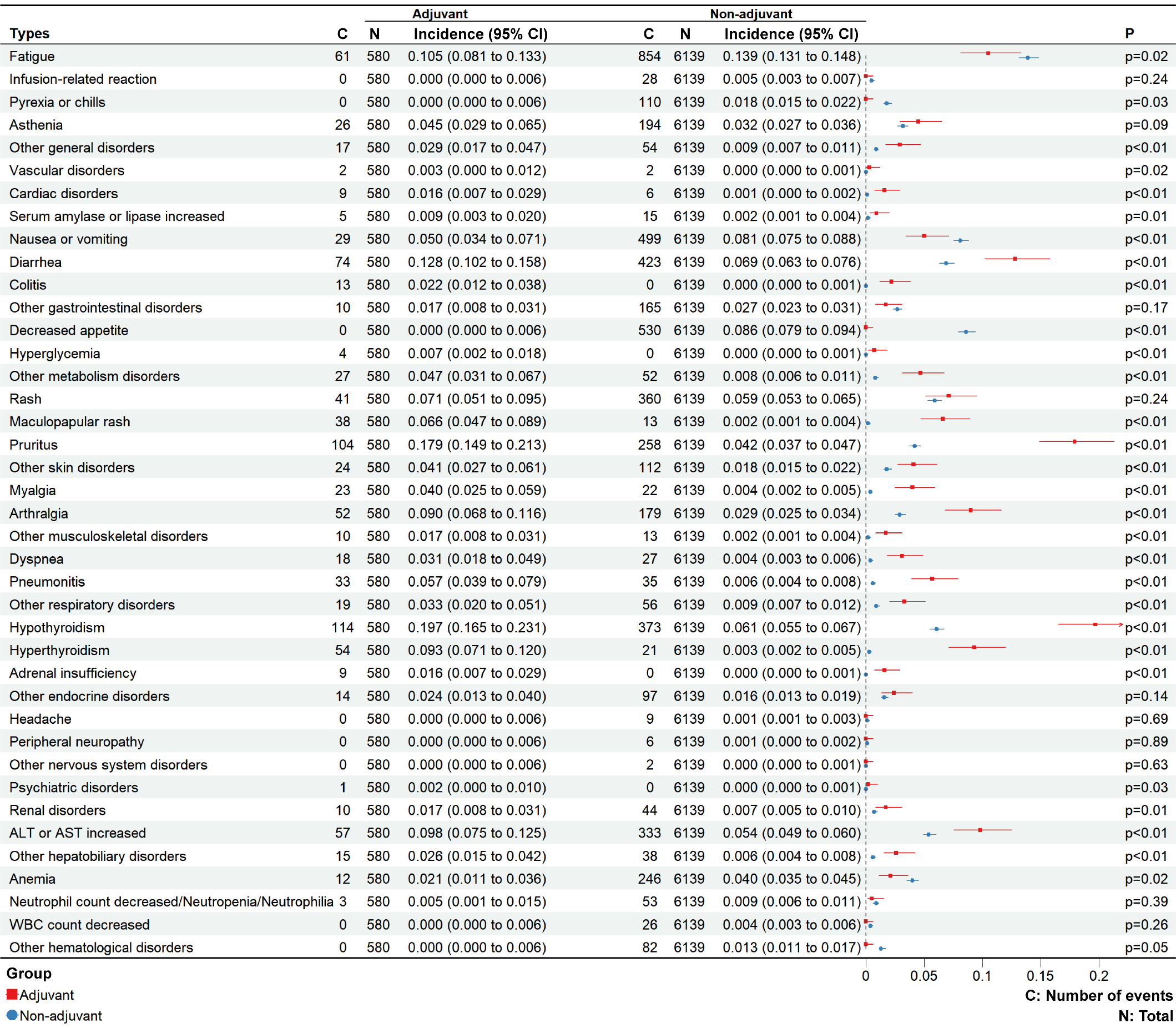


**Figure S14.** **Comparing TRAEs of melanoma patients with single-agent anti-PD-1 as adjuvant and nonadjuvant therapy**


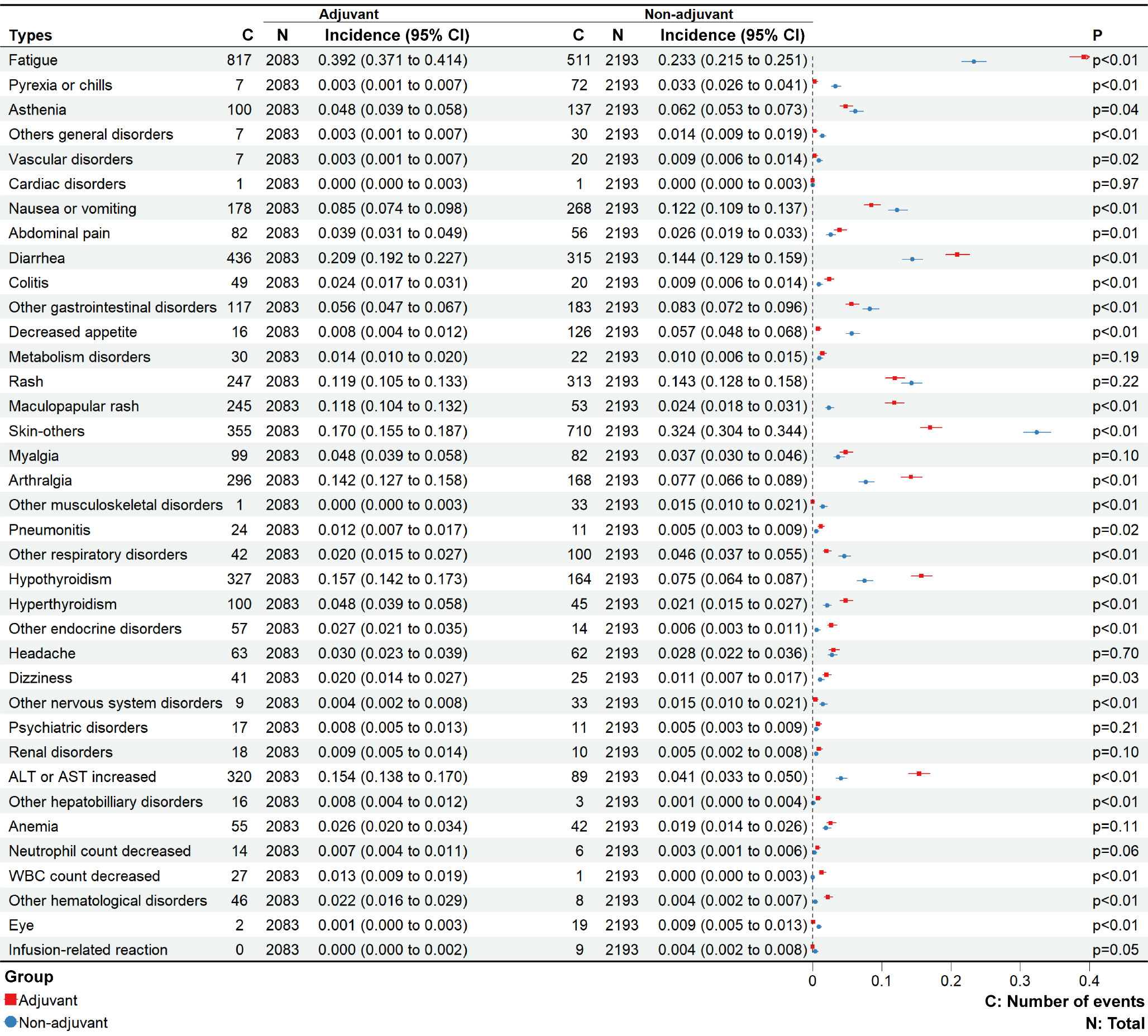


**Figure S15.** **Comparing TRAEs of urothelial carcinoma patients with single-agent anti-PD-L1 as adjuvant and nonadjuvant therapy**


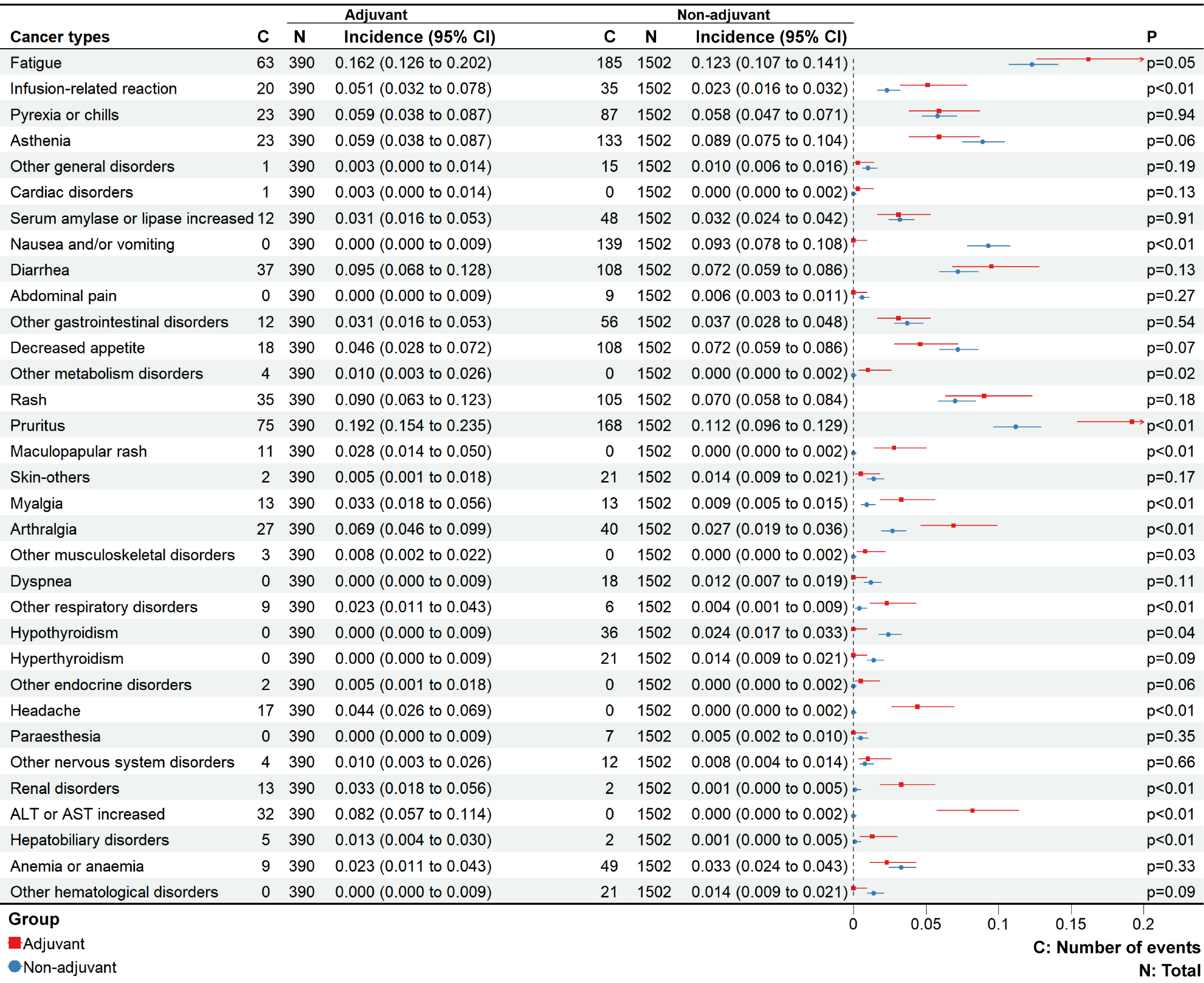


**Figure S16.** **Comparing TRAEs of melanoma patients with single-agent anti-CTLA-4 as adjuvant and nonadjuvant therapy**


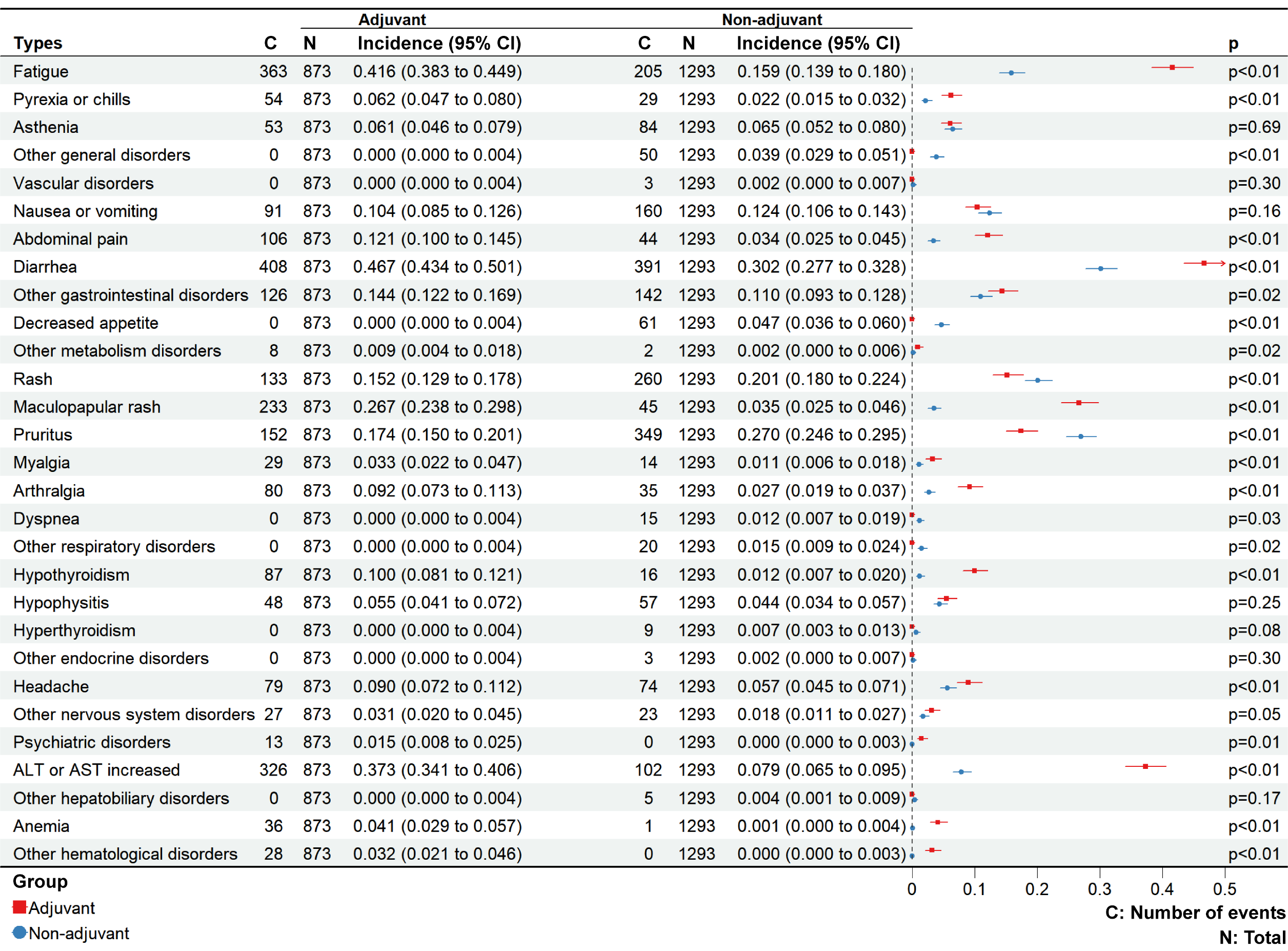


**Figure S17.** **Comparing TRAEs of melanoma patients with single-agent anti-CTLA-4 at different doses.**


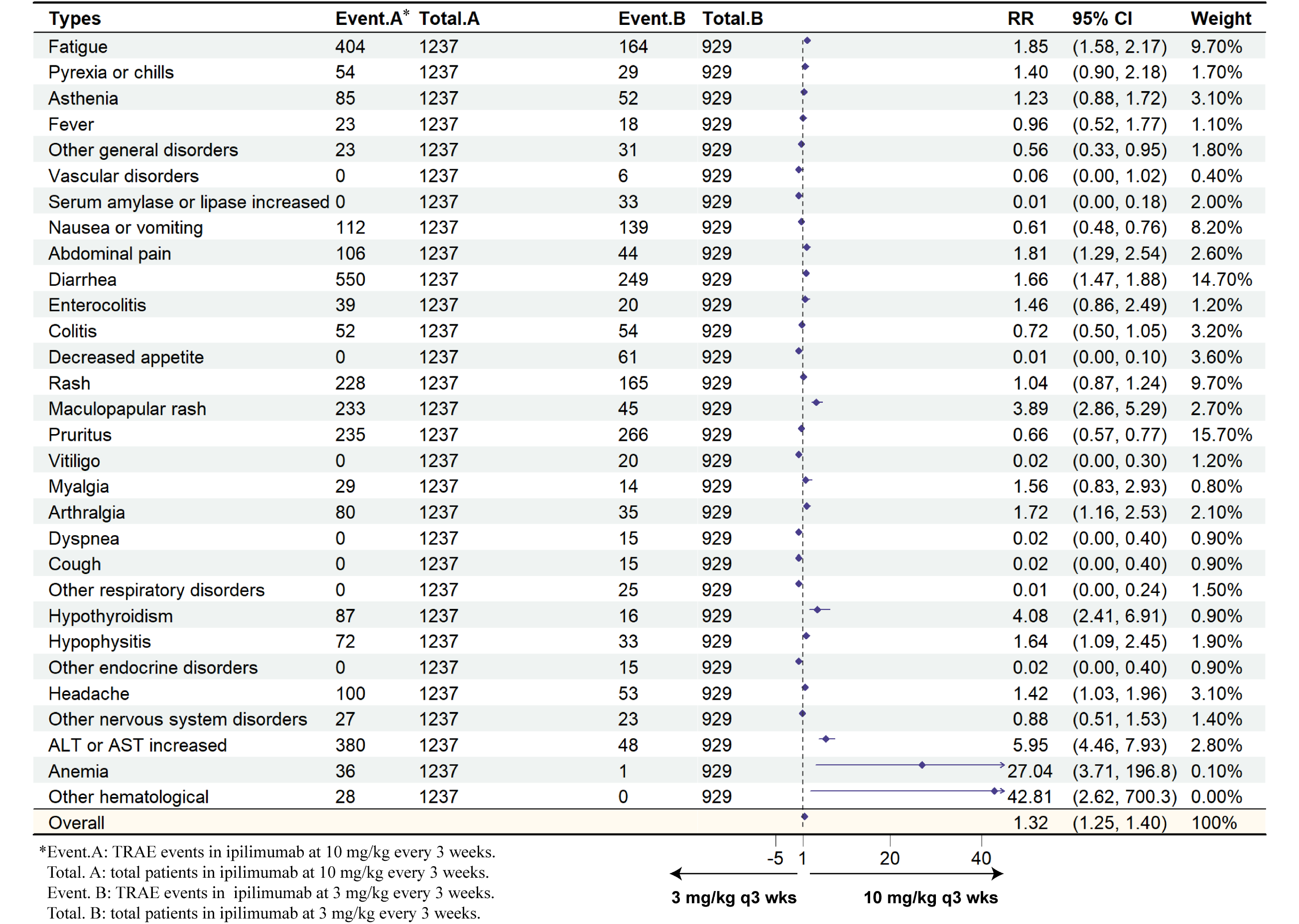


**Table S5. Overall incidences of TRAEs of immune checkpoint inhibitors (ICIs) in different follow-up duration.**

| **ICI target** | **Follow-up duration (month)** | **Incidence** | **95% CI** |
| --- | --- | --- | --- |
| **PD-1** | 0-15 | 0.667 | (0.632,0.699) |
|  | 15-30 | 0.717 | (0.659,0.769) |
|  | 30 and longer | **0.742** | (0.684,0.793) |
| **PD-L1** | 0-15 | 0.630 | (0.563,0.692) |
|  | 15-30 | **0.734** | (0.636,0.814) |
|  | 30 and longer | 0.626 | (0.475,0.756) |
| **CTLA4** | 0-15 | 0.730 | (NA, NA) |
|  | 15-30 | **0.958** | (NA, NA) |
|  | 30 and longer | 0.844 | (0.664,0.936) |

**Table S6. Overall incidences of TRAEs of ICIs in patients with not metastatic vs metastatic cancer patients.**

| **ICI target** | **Not metastatic (95% CI)** | **Metastatic (95% CI)** |
| --- | --- | --- |
| **PD-1** | **0.759** (0.728,0.788) | 0.721 (0.652,0.782) |
| **PD-L1** | **0.711** (0.666,0.752) | 0.601 (0.547,0.653) |
| **CTLA4** | NA | 0.725 (0.633,0.801) |

**Table S7. Overall incidences of TRAEs of ICIs in patients previously untreated vs undergone previous surgery vs previous chemotherapy.**

| **ICI target** | **Untreated**  **(95% CI)** | **Previous surgery**  **(95% CI)** | **Previous chemotherapy**  **(95% CI)** |
| --- | --- | --- | --- |
| **PD-1** | 0.707  (0.636,0.769) | **0.81**  (0.751,0.857) | 0.625  (0.582,0.667) |
| **PD-L1** | 0.565  (0.534,0.597) | **0.759**  (NA, NA) | 0.74  (0.637,0.822) |
| **CTLA4** | 0.862  (NA, NA) | **0.956**  (0.941,0.968) | NA |

**Table S8. Inconsistency Analysis of all grade TRAEs of included studies**

| **ICI target** | **Cancer Type** | **Incidence (95% CI)** | **I^2^*** | **τ^2#^** | **P** |
| --- | --- | --- | --- | --- | --- |
|  |  |  |  |  |  |
| **CTLA-4** | Melanoma | 0.859 (0.723; 0.934) | 97% | 1.0855 | <0.01 |
| **PD-1** | Esophageal or gastroesophageal junction cancer | 0.688 (0.662; 0.712) | 59% | 0.0215 | 0.06 |
|  | Gastric or gastroesophageal cancer | 0.495 (0.461; 0.528) | 79% | 0.0512 | <0.01 |
|  | Head and neck carcinoma | 0.603 (0.571: 0.636) | 0% | 0 | 0.68 |
|  | Hepatocellular carcinoma | 0.640 (0.601; 0.679) | 55% | 0.0010 | 0.14 |
|  | Melanoma | 0.784 (0.738; 0.823) | 91% | 0.1899 | <0.01 |
|  | Lung cancer | 0.681 (0.641; 0.724) | 91% | 0.0139 | <0.01 |
|  | Renal cell carcinoma | 0.789 (0.762; 0.816) | 0% | 0 | 0.85 |
|  | Urothelial carcinoma | 0.640 (0.603; 0.675) | 0% | 0 | 0.40 |
| **PD-L1** | Lung cancer | 0.653 (0.597; 0.706) | 85% | 0.0712 | <0.01 |
|  | Urothelial carcinoma | 0.667 (0.591; 0.743) | 92% | 0.0069 | <0.01 |

*I² quantifies the percentage of total variation across studies that is due to heterogeneity rather than chance.

#τ² represents the variance of the true effects across studies, essentially quantifying the degree of heterogeneity in the effect sizes.

**Figure S18.** **Sensitivity analysis.**


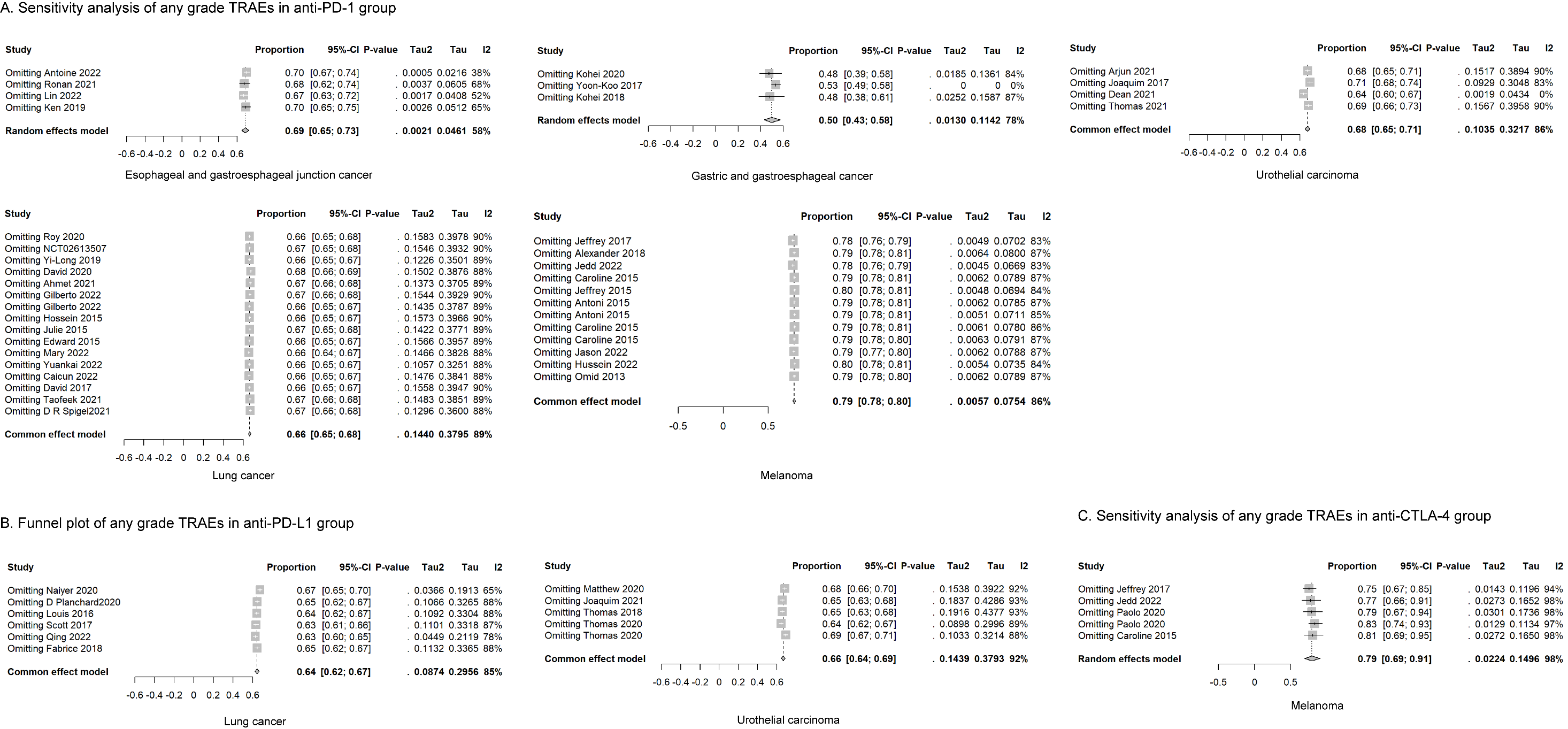


**Figure S19.** **Subgroup analysis.**


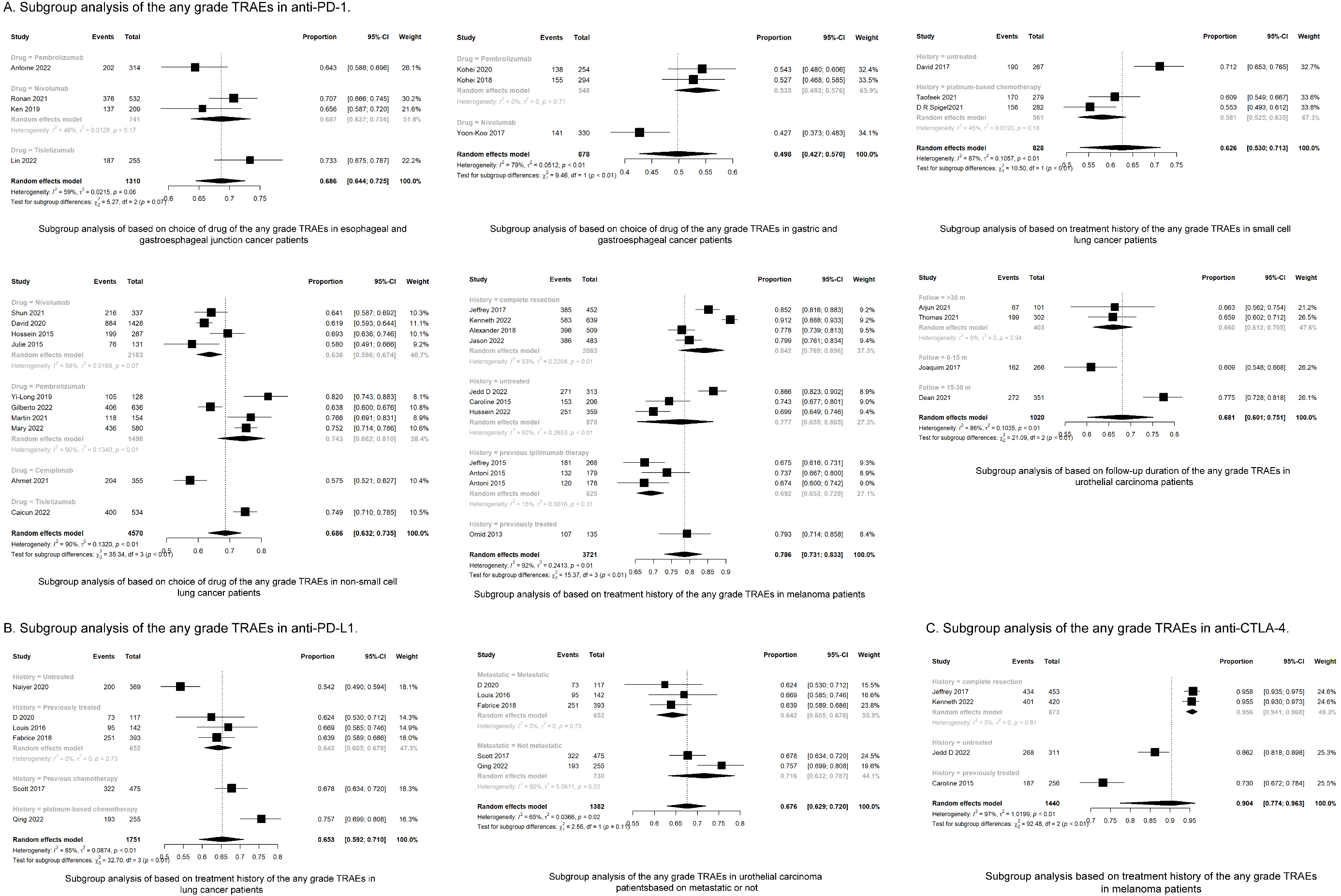


**Reference**

1. Bellmunt J, Hussain M, Gschwend JE, Albers P, Oudard S, Castellano D, et al. Adjuvant atezolizumab versus observation in muscle-invasive urothelial carcinoma (IMvigor010): a multicentre, open-label, randomised, phase 3 trial. Lancet Oncol. 2021;22(4):525-37.

2. Khene ZE, Borchiellini D, Bensalah K. Re: Adjuvant Atezolizumab Versus Placebo for Patients with Renal Cell Carcinoma at Increased Risk of Recurrence Following Resection (IMmotion010): A Multicentre, Randomised, Double-blind, Phase 3 Trial. Eur Urol. 2023;83(5):475-6.

3. Kelly RJ, Ajani JA, Kuzdzal J, Zander T, Van Cutsem E, Piessen G, et al. Adjuvant Nivolumab in Resected Esophageal or Gastroesophageal Junction Cancer. N Engl J Med. 2021;384(13):1191-203.

4. Weber J, Mandala M, Del Vecchio M, Gogas HJ, Arance AM, Cowey CL, et al. Adjuvant Nivolumab versus Ipilimumab in Resected Stage III or IV Melanoma. N Engl J Med. 2017;377(19):1824-35.

5. Bajorin DF, Witjes JA, Gschwend JE, Schenker M, Valderrama BP, Tomita Y, et al. Adjuvant Nivolumab versus Placebo in Muscle-Invasive Urothelial Carcinoma. N Engl J Med. 2021;384(22):2102-14.

6. Grossmann KF, Othus M, Patel SP, Tarhini AA, Sondak VK, Knopp MV, et al. Adjuvant Pembrolizumab versus IFNalpha2b or Ipilimumab in Resected High-Risk Melanoma. Cancer Discov. 2022;12(3):644-53.

7. Eggermont AMM, Blank CU, Mandala M, Long GV, Atkinson V, Dalle S, et al. Adjuvant Pembrolizumab versus Placebo in Resected Stage III Melanoma. N Engl J Med. 2018;378(19):1789-801.

8. Planchard D, Reinmuth N, Orlov S, Fischer JR, Sugawara S, Mandziuk S, et al. ARCTIC: durvalumab with or without tremelimumab as third-line or later treatment of metastatic non-small-cell lung cancer. Ann Oncol. 2020;31(5):609-18.

9. Powles T, Duran I, van der Heijden MS, Loriot Y, Vogelzang NJ, De Giorgi U, et al. Atezolizumab versus chemotherapy in patients with platinum-treated locally advanced or metastatic urothelial carcinoma (IMvigor211): a multicentre, open-label, phase 3 randomised controlled trial. Lancet. 2018;391(10122):748-57.

10. Fehrenbacher L, Spira A, Ballinger M, Kowanetz M, Vansteenkiste J, Mazieres J, et al. Atezolizumab versus docetaxel for patients with previously treated non-small-cell lung cancer (POPLAR): a multicentre, open-label, phase 2 randomised controlled trial. Lancet. 2016;387(10030):1837-46.

11. Galsky MD, Arija JAA, Bamias A, Davis ID, De Santis M, Kikuchi E, et al. Atezolizumab with or without chemotherapy in metastatic urothelial cancer (IMvigor130): a multicentre, randomised, placebo-controlled phase 3 trial. Lancet. 2020;395(10236):1547-57.

12. Pujade-Lauraine E, Fujiwara K, Ledermann JA, Oza AM, Kristeleit R, Ray-Coquard IL, et al. Avelumab alone or in combination with chemotherapy versus chemotherapy alone in platinum-resistant or platinum-refractory ovarian cancer (JAVELIN Ovarian 200): an open-label, three-arm, randomised, phase 3 study. Lancet Oncol. 2021;22(7):1034-46.

13. Powles T, Park SH, Voog E, Caserta C, Valderrama BP, Gurney H, et al. Avelumab Maintenance Therapy for Advanced or Metastatic Urothelial Carcinoma. N Engl J Med. 2020;383(13):1218-30.

14. Barlesi F, Vansteenkiste J, Spigel D, Ishii H, Garassino M, de Marinis F, et al. Avelumab versus docetaxel in patients with platinum-treated advanced non-small-cell lung cancer (JAVELIN Lung 200): an open-label, randomised, phase 3 study. Lancet Oncol. 2018;19(11):1468-79.

15. Qin S, Ren Z, Meng Z, Chen Z, Chai X, Xiong J, et al. Camrelizumab in patients with previously treated advanced hepatocellular carcinoma: a multicentre, open-label, parallel-group, randomised, phase 2 trial. Lancet Oncol. 2020;21(4):571-80.

16. Huang J, Xu J, Chen Y, Zhuang W, Zhang Y, Chen Z, et al. Camrelizumab versus investigator's choice of chemotherapy as second-line therapy for advanced or metastatic oesophageal squamous cell carcinoma (ESCORT): a multicentre, randomised, open-label, phase 3 study. Lancet Oncol. 2020;21(6):832-42.

17. Sezer A, Kilickap S, Gumus M, Bondarenko I, Ozguroglu M, Gogishvili M, et al. Cemiplimab monotherapy for first-line treatment of advanced non-small-cell lung cancer with PD-L1 of at least 50%: a multicentre, open-label, global, phase 3, randomised, controlled trial. Lancet. 2021;397(10274):592-604.

18. Antonia SJ, Villegas A, Daniel D, Vicente D, Murakami S, Hui R, et al. Durvalumab after Chemoradiotherapy in Stage III Non-Small-Cell Lung Cancer. N Engl J Med. 2017;377(20):1919-29.

19. Powles T, van der Heijden MS, Castellano D, Galsky MD, Loriot Y, Petrylak DP, et al. Durvalumab alone and durvalumab plus tremelimumab versus chemotherapy in previously untreated patients with unresectable, locally advanced or metastatic urothelial carcinoma (DANUBE): a randomised, open-label, multicentre, phase 3 trial. Lancet Oncol. 2020;21(12):1574-88.

20. Ferris RL, Haddad R, Even C, Tahara M, Dvorkin M, Ciuleanu TE, et al. Durvalumab with or without tremelimumab in patients with recurrent or metastatic head and neck squamous cell carcinoma: EAGLE, a randomized, open-label phase III study. Ann Oncol. 2020;31(7):942-50.

21. Rizvi NA, Cho BC, Reinmuth N, Lee KH, Luft A, Ahn MJ, et al. Durvalumab With or Without Tremelimumab vs Standard Chemotherapy in First-line Treatment of Metastatic Non-Small Cell Lung Cancer: The MYSTIC Phase 3 Randomized Clinical Trial. JAMA Oncol. 2020;6(5):661-74.

22. Reardon DA, Brandes AA, Omuro A, Mulholland P, Lim M, Wick A, et al. Effect of Nivolumab vs Bevacizumab in Patients With Recurrent Glioblastoma: The CheckMate 143 Phase 3 Randomized Clinical Trial. JAMA Oncol. 2020;6(7):1003-10.

23. Shitara K, Van Cutsem E, Bang YJ, Fuchs C, Wyrwicz L, Lee KW, et al. Efficacy and Safety of Pembrolizumab or Pembrolizumab Plus Chemotherapy vs Chemotherapy Alone for Patients With First-line, Advanced Gastric Cancer: The KEYNOTE-062 Phase 3 Randomized Clinical Trial. JAMA Oncol. 2020;6(10):1571-80.

24. Carbone DP, Reck M, Paz-Ares L, Creelan B, Horn L, Steins M, et al. First-Line Nivolumab in Stage IV or Recurrent Non-Small-Cell Lung Cancer. N Engl J Med. 2017;376(25):2415-26.

25. de Castro G, Jr., Kudaba I, Wu YL, Lopes G, Kowalski DM, Turna HZ, et al. Five-Year Outcomes With Pembrolizumab Versus Chemotherapy as First-Line Therapy in Patients With Non-Small-Cell Lung Cancer and Programmed Death Ligand-1 Tumor Proportion Score >/= 1% in the KEYNOTE-042 Study. J Clin Oncol. 2023;41(11):1986-91.

26. Reck M, Rodriguez-Abreu D, Robinson AG, Hui R, Csoszi T, Fulop A, et al. Five-Year Outcomes With Pembrolizumab Versus Chemotherapy for Metastatic Non-Small-Cell Lung Cancer With PD-L1 Tumor Proportion Score >/= 50. J Clin Oncol. 2021;39(21):2339-49.

27. Adenis A, Kulkarni AS, Girotto GC, de la Fouchardiere C, Senellart H, van Laarhoven HWM, et al. Impact of Pembrolizumab Versus Chemotherapy as Second-Line Therapy for Advanced Esophageal Cancer on Health-Related Quality of Life in KEYNOTE-181. J Clin Oncol. 2022;40(4):382-91.

28. Emens LA, Cruz C, Eder JP, Braiteh F, Chung C, Tolaney SM, et al. Long-term Clinical Outcomes and Biomarker Analyses of Atezolizumab Therapy for Patients With Metastatic Triple-Negative Breast Cancer: A Phase 1 Study. JAMA Oncol. 2019;5(1):74-82.

29. Herbst RS, Garon EB, Kim DW, Cho BC, Perez-Gracia JL, Han JY, et al. Long-Term Outcomes and Retreatment Among Patients With Previously Treated, Programmed Death-Ligand 1‒Positive, Advanced Non‒Small-Cell Lung Cancer in the KEYNOTE-010 Study. J Clin Oncol. 2020;38(14):1580-90.

30. Wolchok JD, Chiarion-Sileni V, Gonzalez R, Grob JJ, Rutkowski P, Lao CD, et al. Long-Term Outcomes With Nivolumab Plus Ipilimumab or Nivolumab Alone Versus Ipilimumab in Patients With Advanced Melanoma. J Clin Oncol. 2022;40(2):127-37.

31. Owonikoko TK, Park K, Govindan R, Ready N, Reck M, Peters S, et al. Nivolumab and Ipilimumab as Maintenance Therapy in Extensive-Disease Small-Cell Lung Cancer: CheckMate 451. J Clin Oncol. 2021;39(12):1349-59.

32. Ferris RL, Blumenschein G, Jr., Fayette J, Guigay J, Colevas AD, Licitra L, et al. Nivolumab for Recurrent Squamous-Cell Carcinoma of the Head and Neck. N Engl J Med. 2016;375(19):1856-67.

33. Kang YK, Boku N, Satoh T, Ryu MH, Chao Y, Kato K, et al. Nivolumab in patients with advanced gastric or gastro-oesophageal junction cancer refractory to, or intolerant of, at least two previous chemotherapy regimens (ONO-4538-12, ATTRACTION-2): a randomised, double-blind, placebo-controlled, phase 3 trial. Lancet. 2017;390(10111):2461-71.

34. Robert C, Long GV, Brady B, Dutriaux C, Maio M, Mortier L, et al. Nivolumab in previously untreated melanoma without BRAF mutation. N Engl J Med. 2015;372(4):320-30.

35. Weber JS, D'Angelo SP, Minor D, Hodi FS, Gutzmer R, Neyns B, et al. Nivolumab versus chemotherapy in patients with advanced melanoma who progressed after anti-CTLA-4 treatment (CheckMate 037): a randomised, controlled, open-label, phase 3 trial. Lancet Oncol. 2015;16(4):375-84.

36. Kato K, Cho BC, Takahashi M, Okada M, Lin CY, Chin K, et al. Nivolumab versus chemotherapy in patients with advanced oesophageal squamous cell carcinoma refractory or intolerant to previous chemotherapy (ATTRACTION-3): a multicentre, randomised, open-label, phase 3 trial. Lancet Oncol. 2019;20(11):1506-17.

37. Lu S, Wang J, Cheng Y, Mok T, Chang J, Zhang L, et al. Nivolumab versus docetaxel in a predominantly Chinese patient population with previously treated advanced non-small cell lung cancer: 2-year follow-up from a randomized, open-label, phase 3 study (CheckMate 078). Lung Cancer. 2021;152:7-14.

38. Borghaei H, Paz-Ares L, Horn L, Spigel DR, Steins M, Ready NE, et al. Nivolumab versus Docetaxel in Advanced Nonsquamous Non-Small-Cell Lung Cancer. N Engl J Med. 2015;373(17):1627-39.

39. Brahmer J, Reckamp KL, Baas P, Crino L, Eberhardt WE, Poddubskaya E, et al. Nivolumab versus Docetaxel in Advanced Squamous-Cell Non-Small-Cell Lung Cancer. N Engl J Med. 2015;373(2):123-35.

40. Motzer RJ, Escudier B, McDermott DF, George S, Hammers HJ, Srinivas S, et al. Nivolumab versus Everolimus in Advanced Renal-Cell Carcinoma. N Engl J Med. 2015;373(19):1803-13.

41. Hamanishi J, Takeshima N, Katsumata N, Ushijima K, Kimura T, Takeuchi S, et al. Nivolumab Versus Gemcitabine or Pegylated Liposomal Doxorubicin for Patients With Platinum-Resistant Ovarian Cancer: Open-Label, Randomized Trial in Japan (NINJA). J Clin Oncol. 2021;39(33):3671-81.

42. Fennell DA, Ewings S, Ottensmeier C, Califano R, Hanna GG, Hill K, et al. Nivolumab versus placebo in patients with relapsed malignant mesothelioma (CONFIRM): a multicentre, double-blind, randomised, phase 3 trial. Lancet Oncol. 2021;22(11):1530-40.

43. Yau T, Park JW, Finn RS, Cheng AL, Mathurin P, Edeline J, et al. Nivolumab versus sorafenib in advanced hepatocellular carcinoma (CheckMate 459): a randomised, multicentre, open-label, phase 3 trial. Lancet Oncol. 2022;23(1):77-90.

44. Ascierto PA, Del Vecchio M, Mackiewicz A, Robert C, Chiarion-Sileni V, Arance A, et al. Overall survival at 5 years of follow-up in a phase III trial comparing ipilimumab 10 mg/kg with 3 mg/kg in patients with advanced melanoma. J Immunother Cancer. 2020;8(1).

45. Powles T, Csoszi T, Ozguroglu M, Matsubara N, Geczi L, Cheng SY, et al. Pembrolizumab alone or combined with chemotherapy versus chemotherapy as first-line therapy for advanced urothelial carcinoma (KEYNOTE-361): a randomised, open-label, phase 3 trial. Lancet Oncol. 2021;22(7):931-45.

46. Finn RS, Ryoo BY, Merle P, Kudo M, Bouattour M, Lim HY, et al. Pembrolizumab As Second-Line Therapy in Patients With Advanced Hepatocellular Carcinoma in KEYNOTE-240: A Randomized, Double-Blind, Phase III Trial. J Clin Oncol. 2020;38(3):193-202.

47. Bellmunt J, de Wit R, Vaughn DJ, Fradet Y, Lee JL, Fong L, et al. Pembrolizumab as Second-Line Therapy for Advanced Urothelial Carcinoma. N Engl J Med. 2017;376(11):1015-26.

48. Garon EB, Rizvi NA, Hui R, Leighl N, Balmanoukian AS, Eder JP, et al. Pembrolizumab for the treatment of non-small-cell lung cancer. N Engl J Med. 2015;372(21):2018-28.

49. Andre T, Shiu KK, Kim TW, Jensen BV, Jensen LH, Punt C, et al. Pembrolizumab in Microsatellite-Instability-High Advanced Colorectal Cancer. N Engl J Med. 2020;383(23):2207-18.

50. Balar AV, Kamat AM, Kulkarni GS, Uchio EM, Boormans JL, Roumiguie M, et al. Pembrolizumab monotherapy for the treatment of high-risk non-muscle-invasive bladder cancer unresponsive to BCG (KEYNOTE-057): an open-label, single-arm, multicentre, phase 2 study. Lancet Oncol. 2021;22(7):919-30.

51. Chan ATC, Lee VHF, Hong RL, Ahn MJ, Chong WQ, Kim SB, et al. Pembrolizumab monotherapy versus chemotherapy in platinum-pretreated, recurrent or metastatic nasopharyngeal cancer (KEYNOTE-122): an open-label, randomized, phase III trial. Ann Oncol. 2023;34(3):251-61.

52. Kuruvilla J, Ramchandren R, Santoro A, Paszkiewicz-Kozik E, Gasiorowski R, Johnson NA, et al. Pembrolizumab versus brentuximab vedotin in relapsed or refractory classical Hodgkin lymphoma (KEYNOTE-204): an interim analysis of a multicentre, randomised, open-label, phase 3 study. Lancet Oncol. 2021;22(4):512-24.

53. Ribas A, Puzanov I, Dummer R, Schadendorf D, Hamid O, Robert C, et al. Pembrolizumab versus investigator-choice chemotherapy for ipilimumab-refractory melanoma (KEYNOTE-002): a randomised, controlled, phase 2 trial. Lancet Oncol. 2015;16(8):908-18.

54. Winer EP, Lipatov O, Im SA, Goncalves A, Munoz-Couselo E, Lee KS, et al. Pembrolizumab versus investigator-choice chemotherapy for metastatic triple-negative breast cancer (KEYNOTE-119): a randomised, open-label, phase 3 trial. Lancet Oncol. 2021;22(4):499-511.

55. Robert C, Schachter J, Long GV, Arance A, Grob JJ, Mortier L, et al. Pembrolizumab versus Ipilimumab in Advanced Melanoma. N Engl J Med. 2015;372(26):2521-32.

56. Cohen EEW, Soulieres D, Le Tourneau C, Dinis J, Licitra L, Ahn MJ, et al. Pembrolizumab versus methotrexate, docetaxel, or cetuximab for recurrent or metastatic head-and-neck squamous cell carcinoma (KEYNOTE-040): a randomised, open-label, phase 3 study. Lancet. 2019;393(10167):156-67.

57. Shitara K, Ozguroglu M, Bang YJ, Di Bartolomeo M, Mandala M, Ryu MH, et al. Pembrolizumab versus paclitaxel for previously treated, advanced gastric or gastro-oesophageal junction cancer (KEYNOTE-061): a randomised, open-label, controlled, phase 3 trial. Lancet. 2018;392(10142):123-33.

58. O'Brien M, Paz-Ares L, Marreaud S, Dafni U, Oselin K, Havel L, et al. Pembrolizumab versus placebo as adjuvant therapy for completely resected stage IB-IIIA non-small-cell lung cancer (PEARLS/KEYNOTE-091): an interim analysis of a randomised, triple-blind, phase 3 trial. Lancet Oncol. 2022;23(10):1274-86.

59. Luke JJ, Rutkowski P, Queirolo P, Del Vecchio M, Mackiewicz J, Chiarion-Sileni V, et al. Pembrolizumab versus placebo as adjuvant therapy in completely resected stage IIB or IIC melanoma (KEYNOTE-716): a randomised, double-blind, phase 3 trial. Lancet. 2022;399(10336):1718-29.

60. Powles T, Tomczak P, Park SH, Venugopal B, Ferguson T, Symeonides SN, et al. Pembrolizumab versus placebo as post-nephrectomy adjuvant therapy for clear cell renal cell carcinoma (KEYNOTE-564): 30-month follow-up analysis of a multicentre, randomised, double-blind, placebo-controlled, phase 3 trial. Lancet Oncol. 2022;23(9):1133-44.

61. Qin S, Chen Z, Fang W, Ren Z, Xu R, Ryoo BY, et al. Pembrolizumab Versus Placebo as Second-Line Therapy in Patients From Asia With Advanced Hepatocellular Carcinoma: A Randomized, Double-Blind, Phase III Trial. J Clin Oncol. 2023;41(7):1434-43.

62. Harrington KJ, Burtness B, Greil R, Soulieres D, Tahara M, de Castro G, Jr., et al. Pembrolizumab With or Without Chemotherapy in Recurrent or Metastatic Head and Neck Squamous Cell Carcinoma: Updated Results of the Phase III KEYNOTE-048 Study. J Clin Oncol. 2023;41(4):790-802.

63. Bang YJ, Ruiz EY, Van Cutsem E, Lee KW, Wyrwicz L, Schenker M, et al. Phase III, randomised trial of avelumab versus physician's choice of chemotherapy as third-line treatment of patients with advanced gastric or gastro-oesophageal junction cancer: primary analysis of JAVELIN Gastric 300. Ann Oncol. 2018;29(10):2052-60.

64. Wu YL, Zhang L, Fan Y, Zhou J, Zhang L, Zhou Q, et al. Randomized clinical trial of pembrolizumab vs chemotherapy for previously untreated Chinese patients with PD-L1-positive locally advanced or metastatic non-small-cell lung cancer: KEYNOTE-042 China Study. Int J Cancer. 2021;148(9):2313-20.

65. Beer TM, Kwon ED, Drake CG, Fizazi K, Logothetis C, Gravis G, et al. Randomized, Double-Blind, Phase III Trial of Ipilimumab Versus Placebo in Asymptomatic or Minimally Symptomatic Patients With Metastatic Chemotherapy-Naive Castration-Resistant Prostate Cancer. J Clin Oncol. 2017;35(1):40-7.

66. Tawbi HA, Schadendorf D, Lipson EJ, Ascierto PA, Matamala L, Castillo Gutierrez E, et al. Relatlimab and Nivolumab versus Nivolumab in Untreated Advanced Melanoma. N Engl J Med. 2022;386(1):24-34.

67. Hamid O, Robert C, Daud A, Hodi FS, Hwu WJ, Kefford R, et al. Safety and tumor responses with lambrolizumab (anti-PD-1) in melanoma. N Engl J Med. 2013;369(2):134-44.

68. Spigel DR, McCleod M, Jotte RM, Einhorn L, Horn L, Waterhouse DM, et al. Safety, Efficacy, and Patient-Reported Health-Related Quality of Life and Symptom Burden with Nivolumab in Patients with Advanced Non-Small Cell Lung Cancer, Including Patients Aged 70 Years or Older or with Poor Performance Status (CheckMate 153). J Thorac Oncol. 2019;14(9):1628-39.

69. Kelley RK, Sangro B, Harris W, Ikeda M, Okusaka T, Kang YK, et al. Safety, Efficacy, and Pharmacodynamics of Tremelimumab Plus Durvalumab for Patients With Unresectable Hepatocellular Carcinoma: Randomized Expansion of a Phase I/II Study. J Clin Oncol. 2021;39(27):2991-3001.

70. Spigel DR, Vicente D, Ciuleanu TE, Gettinger S, Peters S, Horn L, et al. Second-line nivolumab in relapsed small-cell lung cancer: CheckMate 331(☆). Ann Oncol. 2021;32(5):631-41.

71. Shi Y, Wu L, Yu X, Xing P, Wang Y, Zhou J, et al. Sintilimab versus docetaxel as second-line treatment in advanced or metastatic squamous non-small-cell lung cancer: an open-label, randomized controlled phase 3 trial (ORIENT-3). Cancer Commun (Lond). 2022;42(12):1314-30.

72. Zhou Q, Chen M, Jiang O, Pan Y, Hu D, Lin Q, et al. Sugemalimab versus placebo after concurrent or sequential chemoradiotherapy in patients with locally advanced, unresectable, stage III non-small-cell lung cancer in China (GEMSTONE-301): interim results of a randomised, double-blind, multicentre, phase 3 trial. Lancet Oncol. 2022;23(2):209-19.

73. Tewari KS, Monk BJ, Vergote I, Miller A, de Melo AC, Kim HS, et al. Survival with Cemiplimab in Recurrent Cervical Cancer. N Engl J Med. 2022;386(6):544-55.

74. Shen L, Kato K, Kim SB, Ajani JA, Zhao K, He Z, et al. Tislelizumab Versus Chemotherapy as Second-Line Treatment for Advanced or Metastatic Esophageal Squamous Cell Carcinoma (RATIONALE-302): A Randomized Phase III Study. J Clin Oncol. 2022;40(26):3065-76.

75. Zhou C, Huang D, Fan Y, Yu X, Liu Y, Shu Y, et al. Tislelizumab Versus Docetaxel in Patients With Previously Treated Advanced NSCLC (RATIONALE-303): A Phase 3, Open-Label, Randomized Controlled Trial. J Thorac Oncol. 2023;18(1):93-105.
